# Greek Translation and Preliminary Test–Retest Reliability of the AQUA CHILD Questionnaire in Children With Developmental Disabilities

**DOI:** 10.1002/pri.70357

**Published:** 2026-10-08

**Authors:** Stavroula Triantafyllidou, Michaela Christina Tsangala, Eleni Argyroula Tsounia, Chrysoula Moscholouri, Thomas Besios, George Paras, Alfonso Trinidad Morales, Konstantinos Chandolias

**Affiliations:** ^1^ Department of Physiotherapy School of Health Sciences University of Thessaly Lamia Greece; ^2^ Department of Education and Educational Innovation Faculty of Law Education and Humanities Universidad Europea de Madrid Villaviciosa de Odón Madrid Spain; ^3^ Aqualab Research Group Universidad Europea de Madrid Villaviciosa de Odón Madrid Spain; ^4^ Department of Physiotherapy Health Assessment and Quality of Life Research Laboratory School of Health Sciences University of Thessaly Lamia Greece

**Keywords:** child, cross‐cultural adaptation, disability, psychometrics, questionnaires

## Abstract

**Background and Purpose:**

AQUA CHILD is a parent‐proxy questionnaire for pre‐aquatic assessment. This study describes its Greek translation and examines preliminary test–retest agreement, with explicit verification of response coding and the limits of score interpretation.

**Methods:**

In a methodological repeated‐measures study, 47 parents completed the questionnaire 7 days apart. The retained electronic paired files contained 99 of the 102 numbered items on the supplied forms. Swimming responses were analysed categorically; 20 non‐swimming domain summaries were analysed as exploratory raw‐code sums, excluding non‐applicable responses and using complete domain pairs (*n* = 43–47). Intraclass correlation coefficients for absolute agreement, ICC(A, 1), and 95% confidence intervals (CIs), systematic differences, measurement‐error estimates, and participant‐level Bland–Altman analyses were calculated. No overall score was retained.

**Results:**

Swimming‐item exact agreement was 91.5%–97.9% (unweighted kappa, 0.899–0.974). Non‐swimming ICCs ranged from 0.878 to 0.968, with lower 95% CI limits of 0.786–0.942. Only undertaking a single task retained a mean retest increase after Holm adjustment: 0.22 raw‐code points (95% CI, 0.09–0.34; adjusted *p* = 0.019). Baseline domain alpha coefficients ranged from −0.375 to 0.329. At T2, 41 non‐swimming responses from 24 of 47 participants were ‘not relevant’, all ‘not capable’ at T1; none were ‘not relevant’ at T1. Two domain analyses used incomplete item sets. Normal‐theory limits of agreement were sensitive to the discrete, zero‐heavy differences.

**Discussion:**

These estimates describe the retained electronic records. The original paper questionnaires are no longer available, so the accuracy and origin of the 41 transitions could not be independently verified; this unresolved data‐quality issue may bias reliability estimates. Unconfirmed clinical stability, incomplete adaptation records, and missing items further limit inference. The findings do not establish validated domain scales, diagnosis‐specific reliability, or an overall score. Prospective validation with independent source‐data verification remains necessary before individual treatment decisions.

## 1 Introduction

Aquatic physiotherapy is used in paediatric rehabilitation because buoyancy can reduce effective weight bearing, water resistance can provide graded multidirectional loading, and the aquatic environment can support movement practice in a distinctive sensory and motivational context. Reviews suggest potential benefits for selected motor, social, and executive‐function outcomes in children and adolescents with neurodevelopmental or physical disabilities; however, interventions, target populations, and outcome measures remain heterogeneous, which constrains comparison across studies and complicates treatment planning (Ogonowska‐Slodownik et al. 2024; Shariat et al. 2024).

A structured pre‐intervention assessment is therefore important. Direct aquatic performance measures, including the Water Orientation Test of Alyn, Swimming With Independent Measure, and Aquatic Independence Measure, describe observable behaviour or independence in water (Chacham‐Guber et al. 2025; Chandolias et al. 2019, 2021; Tirosh et al. 2008). Such measures do not, by themselves, capture the child's activities and participation across home, school, community, and therapy settings. Parent‐proxy reporting can complement direct observation by identifying assistance needs, participation restrictions, everyday performance, and family priorities that may not be visible during one clinical encounter.

AQUA CHILD was developed as a parent‐proxy pre‐aquatic questionnaire informed by the International Classification of Functioning, Disability and Health for Children and Youth (ICF‐CY). It comprises general information, 21 activity and participation domains, and a summary and goal‐setting component (Frumer et al. 2024; World Health Organization 2007). The development study reported favourable face‐validity and reliability findings, but these cannot be assumed to transfer unchanged across languages, questionnaire versions, or administration methods. Cross‐cultural adaptation addresses semantic, idiomatic, experiential, and conceptual equivalence rather than literal translation alone (Beaton et al. 2000; Wild et al. 2005).

The study aimed to translate AQUA CHILD for Greek‐speaking families and examine 7‐day test–retest reliability. During revision, paired item‐level data and both forms permitted a scoring audit. Analyses therefore address categorical agreement and exploratory domain summaries rather than an overall score. Item‐level analyses, applicability rules, and sensitivity analyses were revision‐stage decisions. Construct validity, measurement invariance, responsiveness, and diagnosis‐specific properties were not evaluated.

## 2 Methods

### 2.1 Study Design

A methodological translation and repeated‐measures test–retest study was conducted. Reporting was informed by COSMIN Reporting Guideline 2.0 and the distinction between reliability and agreement, without implying full COSMIN design compliance (de Vet et al. 2006; Gagnier et al. 2025).

### 2.2 Participants and Setting

A convenience sample of 47 Greek‐speaking parent–child dyads was recruited before planned aquatic therapy. Eligibility comprised age 6–12 years, a developmental or motor disorder, and medical stability sufficient for participation. The same caregiver completed both administrations. Diagnoses were autism spectrum disorder (*n* = 20), cerebral palsy (*n* = 10), Down syndrome (*n* = 5), and another developmental disorder (*n* = 12). No subgroup‐specific analyses were performed. Demographic and treatment summaries were retained from the study report (Table 1) and not recalculated from the item‐response files.

**TABLE 1:** Demographic and clinical characteristics of the study sample (*N* = 47).

Characteristic	Value
Sample	47 parent–child dyads
Boys/girls	30 (63.8%)/17 (36.2%)
Age, years	9.2 (2.1); range 6–12
Height, cm	132.5 (15.3); range 110–158
Body mass, kg	31.8 (8.6); range 20–50
Autism spectrum disorder	20 (42.6%)
Cerebral palsy	10 (21.3%)
Down syndrome	5 (10.6%)
Other developmental disorders	12 (25.5%)
Taking medication	15 (31.9%)
Concurrent physiotherapy	26 (55.3%)
Concurrent occupational therapy	22 (46.8%)
Concurrent speech and language therapy	18 (38.3%)

*Note:* Values are *n* (%) or mean (SD), unless otherwise indicated. Concurrent therapy categories are not mutually exclusive. These are study‐report summaries, not a new demographic analysis of the item‐response files.

Recruitment was pragmatic. A supporting analysis report describes a power calculation, but its prospective specification and the assumptions supporting its ICC calculation could not be verified from a dated protocol or original output. Accordingly, no verified a priori sample‐size justification or post hoc observed‐power claim is made. Interpretation emphasises the 95% CIs and the domain‐specific effective sample sizes (Hoenig and Heisey 2001).

### 2.3 AQUA CHILD Questionnaire and Score Construction

The published development analysis reported 116 categories across 21 domains (Frumer et al. 2024). In contrast, the English and Greek forms supplied for this study contain 102 numbered response items, and each paired data file contains 99 item variables. Variables 13.6 (electric wheelchair or other powered mobility), 21.7 (watching television), and 21.8 (playing with animals) were absent. They were not imputed. Domain 13 and Domain 21 therefore refer only to available‐item subsets (5 of 6 and 6 of 8 items, respectively). The full domain and item mappings are provided in Tables S1–S2.

Response labels were checked in both forms. Swimming codes run from 0 (independent) to 6 (does not swim), with intermediate codes identifying support types (Supporting Information S1). Because these do not establish equal severity intervals, swimming was analysed categorically without summation. In Domains 2–21, 0 denotes independence, 1 support, 2 inability, and 3 non‐applicability. Support types share code 1 and cannot be distinguished electronically. Code 3 was excluded from the sums; no reverse coding or 0–100 transformation was used. Each non‐swimming analysis included only participants whose entire available domain item set was applicable at both visits. Identical items were summed at T1 and T2. These exploratory sums describe support/inability codes, with higher values reflecting more such codes and not developer‐validated scales. Although Frumer et al. (2024) reported a total ICF score, a reproducible overall scoring rule for this version was not established; no overall score was analysed.

### 2.4 Translation Procedure and Documentation

The study account reports permission from the developers, two independent English‐to‐Greek translations (a physiotherapist familiar with paediatric aquatic terminology and a professional translator), reconciliation with a third professional familiar with health terminology, and two independent back‐translations by translators unfamiliar with the source questionnaire. These are the reported stages considered in relation to established adaptation recommendations (Beaton et al. 2000; Wild et al. 2005).

Expert‐committee comparison of the versions and pilot testing with target‐population parents were also reported. However, the retained materials do not identify the exact committee membership, pilot sample size or characteristics, item‐level debriefing findings, or pilot‐to‐study sample overlap. No item‐specific cultural substitution is documented. The process is therefore described as a Greek translation with incompletely documented adaptation procedures, not a fully verified cross‐cultural adaptation or a demonstration of measurement invariance.

### 2.5 Test‐Retest Procedure

The study record describes two administrations 7 days apart and states that no aquatic therapy session or major treatment change occurred between them. T1 denotes the first questionnaire and T2 the retest, rather than pre‐ and post‐intervention measurements. The same parent completed both independently; a researcher clarified instructions when requested. Responses were transcribed from completed paper questionnaires into an electronic spreadsheet. The original completed paper questionnaires are no longer available. Individual administration dates and session logs were unavailable for independent verification. Concurrent physiotherapy, occupational therapy, and speech and language therapy were reported during the study; their attendance and dose specifically between T1 and T2 were not documented. No formal parent‐ or clinician‐rated stability measure was used. Clinical stability was therefore assumed and not directly confirmed.

### 2.6 Statistical Analysis

The original report used IBM SPSS Statistics 25; revision analyses used paired item responses in Python 3.13.5 (NumPy 2.3.5; SciPy 1.17.0). Re‐examination of the retained paired CSV files covered identifiers, item mapping, code ranges, missing fields, and applicability transitions, with reference to the available blank forms and supporting report. Both blank forms include a ‘not relevant’ option. However, the available documentation does not establish how this category was explained on each occasion or independently confirm identical questionnaire versions and original coding/data‐entry procedures at T1 and T2. The cause of the asymmetric pattern could not be established; consistency of electronic variable names does not verify source‐response accuracy. All available items were evaluated using exact agreement and unweighted kappa, retaining non‐applicability as a distinct nominal category (Cohen 1960). Agreement CIs were exact binomial; kappa CIs used 10,000 paired‐participant bootstrap resamples. Non‐swimming sums were summarised descriptively. Paired t tests, mean‐difference CIs, and Holm adjustment across 20 comparisons assessed systematic change (Holm 1979). Sensitivity analyses used exact sign‐flip tests under a symmetry assumption and means of each participant's common applicable items. Detailed formulas, bootstrap settings, exclusions, and sensitivity results are provided in the Supporting Information S1.

Exploratory non‐swimming sums were evaluated with a two‐way random‐effects, absolute‐agreement, single‐measure ICC(A,1), equivalent to ICC(2,1), incorporating systematic occasion differences and random disagreement. F‐based 95% CIs were obtained from participant‐level ANOVA components. Values of 0.50, 0.75, and 0.90 were descriptive reference points; interpretation considered the whole CI and score construction rather than categorising point estimates alone (Koo and Li 2016).

Agreement SEM was the square root of residual variance plus non‐negative occasion variance; MDC95 = 1.96 × √2 × SEM. These model‐based individual error indices are not thresholds for group means or clinically important changes (de Vet et al. 2006; Weir 2005). Participant‐level difference‐versus‐mean plots and nominal normal‐theory limits (mean difference ± 1.96 × SD of differences) were calculated directly (Bland and Altman 1986). Limit uncertainty, empirical quantiles, zero differences, observations outside limits, and magnitude‐related diagnostics are supplied. Normal limits are descriptive for discrete differences; no reconstructed aggregate limits or simulated observations were used.

Following code verification, alpha was calculated at both administrations in the same complete‐case non‐swimming samples. It is a covariance diagnostic, not a proof of unidimensionality, validity, or temporal reliability (Tavakol and Dennick 2011). No overall or swimming alpha was interpreted. All tests were two‐sided with nominal significance level 0.05 and the stated multiplicity adjustments.

### 2.7 AI‐Assisted Revision

ChatGPT (OpenAI) assisted revision drafting, analysis‐code development, and consistency checking. Numerical results were computed by executing the supplied Python code on the paired CSV files; no participant responses were generated. Interpretation and submission approval remain the authors' responsibility.

### 2.8 Ethical Considerations

The study account reports approval by the institutional research ethics committee; identifying approval details are provided on the separate title page for anonymous review. Written informed consent was obtained from the participating parents. Participation was voluntary, withdrawal was permitted without consequence, and confidentiality was maintained in accordance with the Declaration of Helsinki.

## 3 Results

### 3.1 Translation and Data Audit

Reported pilot feedback described understandable wording and perceived questionnaire length, but its frequency and representativeness cannot be assessed without pilot‐level documentation. The two data files contain 47 matching participant identifiers and 99 item variables, without blank or out‐of‐range values. However, the three form items have no data columns. All 41 non‐swimming ‘not relevant’ entries (code 3) occurred at T2 in 24 of 47 participants (51.1%), each following ‘not capable’ (code 2) at T1; none occurred at T1. These recorded applicability changes were excluded from numerical scoring rather than interpreted as improvement. Because the original completed paper questionnaires are no longer available, neither the accuracy of these particular transitions nor their cause could be independently verified.

### 3.2 Participant Characteristics

The children had a mean age of 9.2 years (SD 2.1; range, 6–12); 30 (63.8%) were boys and 17 (36.2%) were girls. Mean height was 132.5 cm (SD 15.3; range, 110–158) and mean body weight was 31.8 kg (SD 8.6; range, 20–50). Fifteen children (31.9%) were taking medication. Concurrent therapies included physiotherapy for 26 children (55.3%), occupational therapy for 22 (46.8%), and speech and language therapy for 18 (38.3%); categories were not mutually exclusive (Table 1).

### 3.3 Test–Retest Agreement, Measurement Error, and Systematic Change

The complete‐case non‐swimming analyses included 43–47 pairs per domain. ICCs ranged from 0.878 (walking; 95% CI, 0.786–0.932) to 0.968 (engagement in play; 95% CI, 0.942–0.982). Lower CI limits ranged from 0.786 to 0.942. These estimates describe 18 complete non‐swimming domains and two available‐item subsets, not all 21 complete domains. Agreement SEM ranged from 0.21 to 0.65 raw‐code points and model‐based MDC95 from 0.58 to 1.79 points (Tables 2 and 3).

**TABLE 2:** Exploratory non‐swimming domain summaries and systematic change.

Domain	Items	*n*	T1 mean (SD)	T2 mean (SD)	T2−T1 [95% CI]	Raw p	Holm p
2. Acquiring skills	3	46	2.41 (1.29)	2.30 (1.19)	−0.11 [−0.26, 0.05]	0.168	1.000
3. Focusing attention	2	46	1.59 (1.11)	1.63 (1.12)	0.04 [−0.04, 0.13]	0.323	1.000
4. Solving problems	4	44	3.55 (1.52)	3.43 (1.61)	−0.11 [−0.25, 0.02]	0.096	1.000
5. Making decisions	3	44	2.84 (1.08)	2.82 (1.08)	−0.02 [−0.18, 0.13]	0.767	1.000
6. Undertaking a single task	3	46	2.52 (1.22)	2.74 (1.32)	0.22 [0.09, 0.34]	< 0.001	0.019
7. Managing one's own behaviour	5	47	4.30 (1.79)	4.43 (1.86)	0.13 [−0.05, 0.31]	0.160	1.000
8. Conversation	4	44	3.77 (1.52)	3.75 (1.59)	−0.02 [−0.16, 0.12]	0.743	1.000
9. Changing basic body positions	9	45	8.27 (2.08)	8.44 (2.04)	0.18 [−0.03, 0.38]	0.088	1.000
10. Maintaining a body position	6	44	5.61 (1.48)	5.66 (1.60)	0.05 [−0.14, 0.23]	0.623	1.000
11. Hand and arm use	4	44	3.23 (1.36)	3.30 (1.49)	0.07 [−0.08, 0.22]	0.372	1.000
12. Walking	8	43	7.23 (1.69)	7.09 (2.00)	−0.14 [−0.42, 0.14]	0.323	1.000
13. Moving around [subset]	5	47	4.26 (1.82)	4.26 (1.89)	0.00 [−0.15, 0.15]	1.000	1.000
14. Moving around in different locations	4	46	3.33 (1.28)	3.30 (1.41)	−0.02 [−0.19, 0.15]	0.799	1.000
15. Caring for body parts	5	43	4.47 (1.39)	4.56 (1.52)	0.09 [−0.07, 0.25]	0.253	1.000
16. Dressing	8	46	6.98 (1.94)	6.96 (2.00)	−0.02 [−0.23, 0.19]	0.837	1.000
17. Basic interpersonal interactions	4	45	3.38 (1.64)	3.44 (1.67)	0.07 [−0.15, 0.28]	0.538	1.000
18. Informal relationships with peers	3	47	2.87 (1.30)	3.02 (1.31)	0.15 [−0.01, 0.31]	0.070	1.000
19. Family relationships	4	46	3.61 (1.39)	3.61 (1.42)	0.00 [−0.14, 0.14]	1.000	1.000
20. Engagement in play	5	45	4.53 (1.79)	4.53 (1.91)	0.00 [−0.14, 0.14]	1.000	1.000
21. Recreation and leisure [subset]	6	43	5.84 (2.05)	5.77 (2.08)	−0.07 [−0.29, 0.15]	0.519	1.000

*Note:* Raw‐code sums use 0 = independent, 1 = support, and 2 = not capable. Code 3 = not relevant is excluded; *n* denotes complete domain pairs. Higher sums represent more support/inability codes, not better function. Domains 13 and 21 contain only 5 of 6 and 6 of 8 items, respectively. Change = T2 − T1. CIs are unadjusted 95% mean‐difference intervals; Holm *p* values cover 20 comparisons. No overall score or swimming sum is included.

**TABLE 3:** ICCs, model‐based agreement error, and exploratory internal consistency of non‐swimming summaries.

Domain	*n*	ICC(A,1) [95% CI]	SEM	MDC95	Alpha T1	Alpha T2
2. Acquiring skills	46	0.909 [0.841, 0.948]	0.38	1.04	0.189	−0.208
3. Focusing attention	46	0.965 [0.938, 0.980]	0.21	0.58	0.296	0.245
4. Solving problems	44	0.958 [0.924, 0.977]	0.32	0.89	0.211	0.269
5. Making decisions	44	0.893 [0.812, 0.940]	0.36	0.99	−0.262	−0.325
6. Undertaking a single task	46	0.934 [0.849, 0.968]	0.33	0.91	0.216	0.282
7. Managing one's own behaviour	47	0.943 [0.900, 0.968]	0.44	1.21	0.329	0.304
8. Conversation	44	0.958 [0.924, 0.977]	0.32	0.90	0.150	0.191
9. Changing basic body positions	45	0.943 [0.897, 0.968]	0.49	1.37	−0.090	−0.264
10. Maintaining a body position	44	0.923 [0.864, 0.957]	0.43	1.19	−0.375	−0.236
11. Hand and arm use	44	0.939 [0.891, 0.966]	0.35	0.98	−0.087	0.033
12. Walking	43	0.878 [0.786, 0.932]	0.65	1.79	−0.283	0.023
13. Moving around [subset]	47	0.963 [0.935, 0.979]	0.36	1.00	0.309	0.351
14. Moving around in different locations	46	0.910 [0.843, 0.949]	0.41	1.13	−0.008	0.077
15. Caring for body parts	43	0.934 [0.882, 0.964]	0.37	1.04	−0.241	−0.060
16. Dressing	46	0.935 [0.886, 0.964]	0.51	1.40	0.048	0.043
17. Basic interpersonal interactions	45	0.907 [0.837, 0.948]	0.51	1.41	0.186	0.180
18. Informal relationships with peers	47	0.906 [0.836, 0.947]	0.40	1.11	0.083	0.046
19. Family relationships	46	0.945 [0.903, 0.969]	0.33	0.92	0.194	0.180
20. Engagement in play	45	0.968 [0.942, 0.982]	0.34	0.93	0.130	0.289
21. Recreation and leisure [subset]	43	0.943 [0.897, 0.968]	0.50	1.38	0.323	0.292

*Note:* ICC(A, 1) denotes the two‐way random‐effects, absolute‐agreement, single‐measure coefficient; CIs are F‐based 95% intervals. SEM includes residual and non‐negative occasion variance; MDC95 = 1.96 × √2 × SEM. Error indices concern individual repeated scores under the model, not group means or clinically important change. Alpha coefficients use the same complete‐case item set at T1 and T2 and do not establish unidimensionality. Domains 13 and 21 are available‐item subsets. Negative occasion‐variance estimates were set to zero for SEM in 12 of 20 analyses. ICC point estimates and CIs retain the conventional ANOVA calculation; truncation affects only the stated error index.

For the four swimming items, exact agreement was 91.5%–97.9% and unweighted kappa was 0.899–0.974 (Table 4). Across all 99 available items, exact agreement ranged from 80.9% to 100% and kappa from 0.715 to 1.000 (Table S3). The participant‐level Bland–Altman analyses for non‐swimming sums are presented in Tables S4–S5 and Supporting Information S1: Figures S1–S20. Differences were discrete and frequently zero; the observed coverage of normal‐theory limits ranged from 77.8% to 100%. No proportional‐bias or heteroscedasticity diagnostic remained significant after adjustment within its 20‐test family, but the sample and restricted differences limited the sensitivity of these tests.

**TABLE 4:** Categorical agreement for swimming items (*N* = 47).

Swimming item	Exact pairs	Agreement % [95% CI]	Unweighted kappa [95% CI]
1.1. Backstroke	45/47	95.7 [85.5, 99.5]	0.949 [0.869, 1.000]
1.2. Crawl stroke	43/47	91.5 [79.6, 97.6]	0.899 [0.796, 0.975]
1.3. Breaststroke	45/47	95.7 [85.5, 99.5]	0.950 [0.871, 1.000]
1.4. Another stroke	46/47	97.9 [88.7, 99.9]	0.974 [0.920, 1.000]

*Note:* Exact agreement is reported with exact binomial 95% CIs. Kappa is unweighted; its 95% CIs are paired‐participant bootstrap percentile intervals (10,000 resamples). Codes identify response categories and are not treated as equally spaced severity grades. The source form codes 0 = independent and 6 = does not swim. Codes 1–5 distinguish support categories. No swimming sum, reverse scoring, or metric 0–100 conversion is analysed.

Undertaking a single task (Domain 6) was the only mean difference retained after Holm adjustment: T1, 2.52 (SD 1.22); T2, 2.74 (SD 1.32); difference, 0.22 points (95% CI, 0.09–0.34; unadjusted *p* = 0.000956; adjusted *p* = 0.019; *n* = 46). Ten participants had a one‐point increase and 36 had no change. The exact sign‐flip sensitivity test also retained this finding (adjusted *p* = 0.039). Higher raw‐code sums indicate more support/inability codes, not improvement. The common‐applicable‐item sensitivity analysis again identified only Domain 6 after adjustment (Table S6). Neither statistical significance nor non‐significance establishes clinical importance or individual stability.

### 3.4 Exploratory Internal Consistency

After the exclusion of non‐applicable responses, T1 alpha coefficients ranged from −0.375 to 0.329; seven of 20 were negative. T2 coefficients ranged from −0.325 to 0.351 (Table 3). Thus, correcting the coding framework did not resolve the weak item covariance. No claim of adequate internal consistency or unidimensional measurement is supported for these sums.

## 4 Discussion

The analysis identifies substantial short‐term agreement in the retained electronic Greek AQUA CHILD records, but does not independently establish the accuracy of the original responses. Swimming is represented by categorical item agreement. Non‐swimming ICCs concern exploratory raw‐code sums in complete domain pairs; two are incomplete‐item subsets. No overall aggregate score was analysed because its interpretation and construction were not adequately supported. These distinctions matter: reproducibility of a recorded response is not equivalent to validation of a multidomain functional scale. The present results support further evaluation of the questionnaire, not unrestricted adoption of numerical scores for treatment decisions.

The reported translation sequence included forward translation, reconciliation, back‐translation, expert review, and parent pilot testing, consistent in outline with adaptation guidance (Beaton et al. 2000; Wild et al. 2005). Nevertheless, knowing the sequence is not equivalent to documenting its execution. The missing expert roster, pilot characteristics, and item‐level debriefing record prevent assessment of whose perspectives were represented and how comprehension problems were resolved. Accordingly, these findings concern Greek translation and preliminary reliability, not demonstrated cultural equivalence. The available Greek wording is a starting point for a prospectively documented evaluation, not evidence that cognitive debriefing or cross‐cultural measurement invariance has been established.

The ICC estimates should be read with their CIs. For example, walking had an estimate of 0.878 but a lower limit of 0.786, whereas engagement in play had an estimate of 0.968 with a lower limit of 0.942. Such intervals convey uncertainty more usefully than a tally of ‘excellent’ point estimates or an observed‐power calculation. Reliability also depends on between‐person heterogeneity: variation between diagnoses can increase the ICC even when clinically relevant within‐person disagreement remains (de Vet et al. 2006). The sample included only 5–20 children in each named diagnostic category. It cannot establish reliability separately in autism spectrum disorder, cerebral palsy, Down syndrome, or other disorders, nor equivalence across these groups. Greek studies of WOTA and SWIM address different measures and rater structures and cannot validate AQUA CHILD score construction (Chandolias et al. 2019, 2021).

The Domain 6 retest shift is not contradicted by its high ICC. A sample can preserve between‐child ordering while showing a systematic occasion effect. Here, ten one‐point increases produced a positive mean change, and the sensitivity analysis for discrete differences supported this finding. We do not compare that group mean with MDC95: the former concerns a systematic difference across the sample, whereas the latter is a model‐based threshold for an individual's repeated scores. Nor do we label the shift in clinical deterioration. Familiarisation, response reconsideration, changes in reported applicability, concurrent treatment, and short‐term fluctuation remain possible explanations. The data do not distinguish these mechanisms, and the absence of aquatic treatment does not by itself establish stability in everyday functioning.

Low and negative alpha coefficients persisted after non‐applicable responses were excluded. However, this does not identify a single cause of the weak covariance. Negative alpha signals negative average covariance among the included items; broad content alone is not a sufficient explanation for accepting a sum as a coherent scale (Tavakol and Dennick 2011). The original development study reported markedly higher domain alpha values, but evaluated a different item configuration and administration comparison (Frumer et al. 2024). Without verified dimensionality and a justified measurement model, neither the original estimates nor the present high ICCs establish valid summed scales. Item‐level information may be clinically informative even when a numerical sum lacks internal‐consistency support.

The applicability pattern remains an unresolved data‐quality concern: 41 ‘not capable’–to–‘not relevant’ transitions affected 24 of 47 participants, with no non‐swimming ‘not relevant’ responses at T1. One possible, but unverified, explanation is that some parents interpreted the distinction between ‘not capable’ and ‘not relevant’ differently across administrations. An activity not performed by the child might have been classified as inability on one occasion and non‐applicability on the other, without an actual change in the child's functioning. The retained records provide no direct evidence establishing such a reinterpretation. This hypothesis does not establish why ‘not relevant’ responses occurred exclusively at T2 and does not resolve the data‐quality concern. Without the original completed paper questionnaires, the retained records cannot distinguish changed response interpretation, administration, instructions, or coding/data‐entry procedures from genuine changes in applicability. Counting code 3 as impairment conflates irrelevance with inability; recoding it to 2 assumes the original answer. Neither is justified. Complete‐domain exclusions avoid summing non‐applicable codes but may select more consistent pairs and overestimate reliability; the actual direction and magnitude of any bias are unknown. Item‐level agreement retains these recorded disagreements and may also be affected by transcription error. The common‐applicable‐item sensitivity analysis cannot verify original responses or remove this uncertainty. Missing variables were not replaced with zeros.

For physiotherapy practice, the instrument's broad questions may help organise discussion of activity, participation, assistance, and family priorities alongside direct examination. That potential use should be separated from validated outcome measurement. No minimal important change, responsiveness, diagnostic discrimination, or individual treatment‐response rule has been established here. The difference plots improve transparency, but their discrete, zero‐heavy distributions mean that nominal normal‐theory limits do not cover 95% of observed differences. The empirical ranges and limit uncertainty should therefore be examined together, rather than converting the reported SEM, MDC95, or limits into clinical cut‐offs.

Although this study concerns the Greek translation of AQUA CHILD, the methodological issues identified may be relevant to the adaptation of rehabilitation questionnaires across languages and health‐care settings. Translation alone does not establish measurement equivalence, and differences in the interpretation of response categories such as ‘not capable’ and ‘not relevant’ may influence reliability estimates. Future cross‐cultural validation studies should prospectively document adaptation procedures, retain source records, verify data entry, and examine whether response categories are understood consistently across administrations.

### 4.1 Strengths and Limitations

Strengths include paired item‐level analysis, respondent continuity, code‐label checking against both blank forms, disclosure of absent items and applicability changes, and separate assessment of agreement, reliability, and error. A major limitation is that the responses were transcribed from paper questionnaires that are no longer available. The accuracy of the 41 asymmetric transitions could not therefore be independently verified. The estimates are conditional on the retained electronic records, and a possible contribution from transcription or coding error cannot be excluded. Additional limitations include convenience sampling, domain‐specific exclusions, two occasions, unconfirmed stability, incomplete adaptation records, and an unverifiable prospective sample‐size plan. Concurrent therapies were reported, but intervening exposure could not be quantified.

### 4.2 Future Research

Future studies should retain original response records and a documented data‐entry audit trail, verify electronic entries against those sources, include all questionnaire items, and obtain version‐specific developer scoring instructions. Prospective studies should document cognitive debriefing, justify any summed measurement model, and plan ICC precision. Such debriefing should specifically examine how parents distinguish inability from non‐applicability. Larger stratified samples and direct stability ratings are needed before diagnosis‐specific claims. Construct validity and responsiveness should then be evaluated against prespecified hypotheses and external measures of meaningful change.

## 5 Conclusions

The retained electronic Greek AQUA CHILD records showed preliminary short‐term reproducibility in this heterogeneous convenience sample. The accuracy and origin of the 41 ‘not capable’–to–‘not relevant’ transitions could not be independently verified because the original paper questionnaires are no longer available. This unresolved data‐quality issue limits interpretation of the reliability estimates. The findings do not establish a validated overall score, coherent domain scales, complete‐questionnaire reliability, or diagnosis‐specific measurement properties. Prospective validation with retained source records and independent response verification is required before numerical changes are used to make individual clinical decisions.

## 6 Implications for Physiotherapy Practice

The Greek questionnaire may help structure discussion of everyday activity, participation, assistance, and family priorities alongside direct aquatic assessment.

The reported agreement applies to the available item responses and explicitly defined exploratory summaries, not to an authorised overall functional score or to every diagnostic group.

Neither group mean change nor model‐based MDC95 should be used alone to infer an individual treatment effect; applicability, clinical stability, validity, and responsiveness require further verification.

## Author Contributions

Conceptualisation and supervision: Konstantinos Chandolias. Methodology: Stavroula Triantafyllidou, Michaela Christina Tsaggala, Eleni Argyroula Tsounia, Thomas Besios, George Paras, Alfonso Trinidad Morales, and Konstantinos Chandolias. Investigation and data collection: Stavroula Triantafyllidou and Michaela Christina Tsaggala. Formal analysis: Konstantinos Chandolias, Thomas Besios, and George Paras. Writing – original draft: Stavroula Triantafyllidou and Michaela Christina Tsaggala. Writing – review and editing: Eleni Argyroula Tsounia, Chrysoula Moscholouri, Thomas Besios, George Paras, Alfonso Trinidad Morales, and Konstantinos Chandolias.

## Funding

The authors have nothing to report.

## Ethics Statement

The study was approved by the Institutional Ethics Committee of the University of Thessaly (approval no. 25522/25/ΤΦΣΚ).

## Consent

Written informed consent was obtained from all participating parents. Participation was voluntary and confidentiality was maintained.

## Conflicts of Interest

The authors declare no conflicts of interest.

## Supporting information


Supporting Information S1

**Table S1:** Domain structure and available data.

**Table S2:** Item‐to‐domain crosswalk.

**Table S3:** Item‐level categorical agreement.

**Table S4:** Direct participant‐level difference summaries and limits of agreement.

**Table S5:** Difference‐distribution and magnitude diagnostics.

**Table S6:** (A) Common‐applicable‐item sensitivity analysis. (B) Exact sign‐flip sensitivity of the fixed‐item paired differences.

## Data Availability

De‐identified paired item responses were available for the present reanalysis. Numerical summaries and the analysis method are provided in the Supplementary Material. Requests for participant‐level electronic data and executable code may be directed to the corresponding author, subject to applicable consent, institutional ethics, and confidentiality requirements. The electronic records were transcribed from completed paper questionnaires, which are no longer available. They therefore cannot be supplied for independent source verification, and the accuracy and cause of the 41 ‘not capable’–to–‘not relevant’ transitions remain unverified. Three numbered form items are absent from the available electronic files; independent administration and data‐entry source records were not available for this audit.
