# Supplementary material for: Greek Translation and Preliminary Test–Retest Reliability of the AQUA CHILD Questionnaire in Children With Developmental Disabilities
Source: Physiother Res Int. 2026 Oct 8;31(4):e70357. doi: 10.1002/pri.70357 (PMC13648008; doi:10.1002/pri.70357)
Supplement: Supplementary file 1 [file PRI-31-e70357-s006.pdf]

Figure S1. Domain 2: Acquiring skills

n = 46 paired observations | 3 items | no jitter or simulated observations

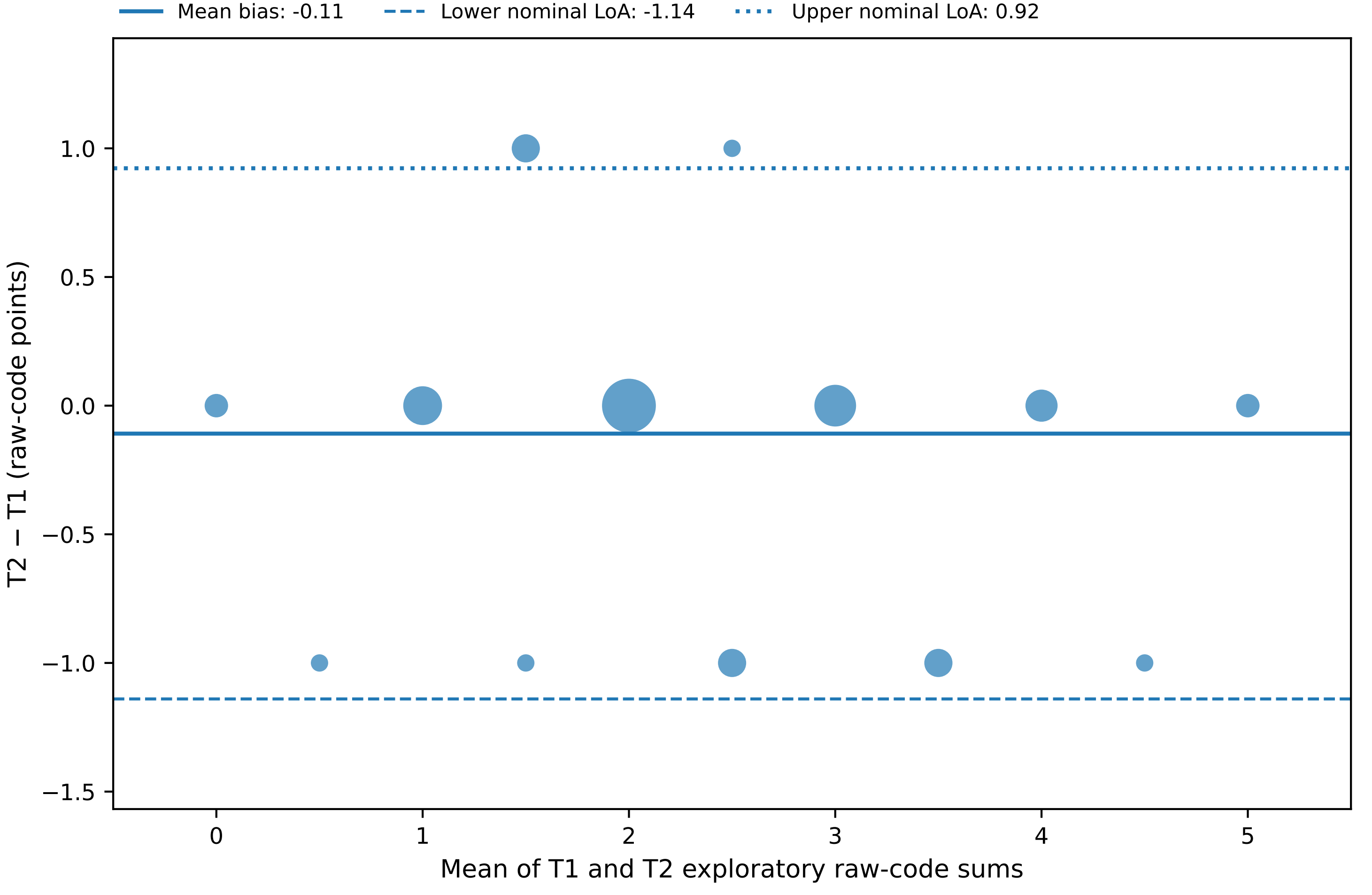

Each marker represents one or more observed pairs at identical coordinates; marker area is proportional to the number of pairs (maximum 12). Complete domain pairs were used, excluding non-applicable responses at either visit. Limits are mean difference ± 1.96 × SD of the differences. Discrete, zero-heavy differences mean that these normal-theory limits need not cover 95% of observed pairs. They are not validated clinical decision thresholds.

Figure S2. Domain 3: Focusing attention

n = 46 paired observations | 2 items | no jitter or simulated observations

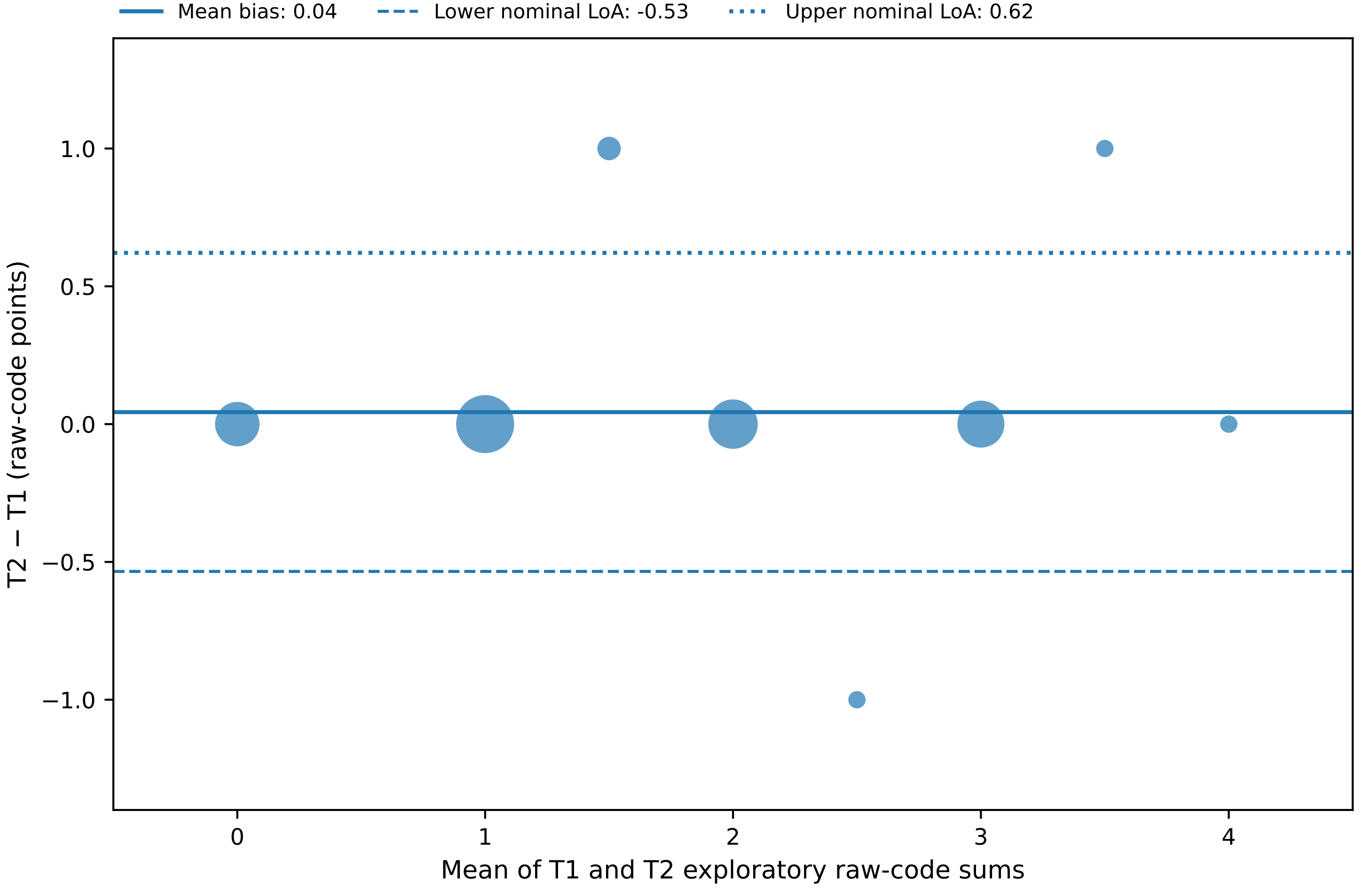

Each marker represents one or more observed pairs at identical coordinates; marker area is proportional to the number of pairs (maximum 14). Complete domain pairs were used, excluding non-applicable responses at either visit. Limits are mean difference ± 1.96 × SD of the differences. Discrete, zero-heavy differences mean that these normal-theory limits need not cover 95% of observed pairs. They are not validated clinical decision thresholds.

Figure S3. Domain 4: Solving problems

n = 44 paired observations | 4 items | no jitter or simulated observations

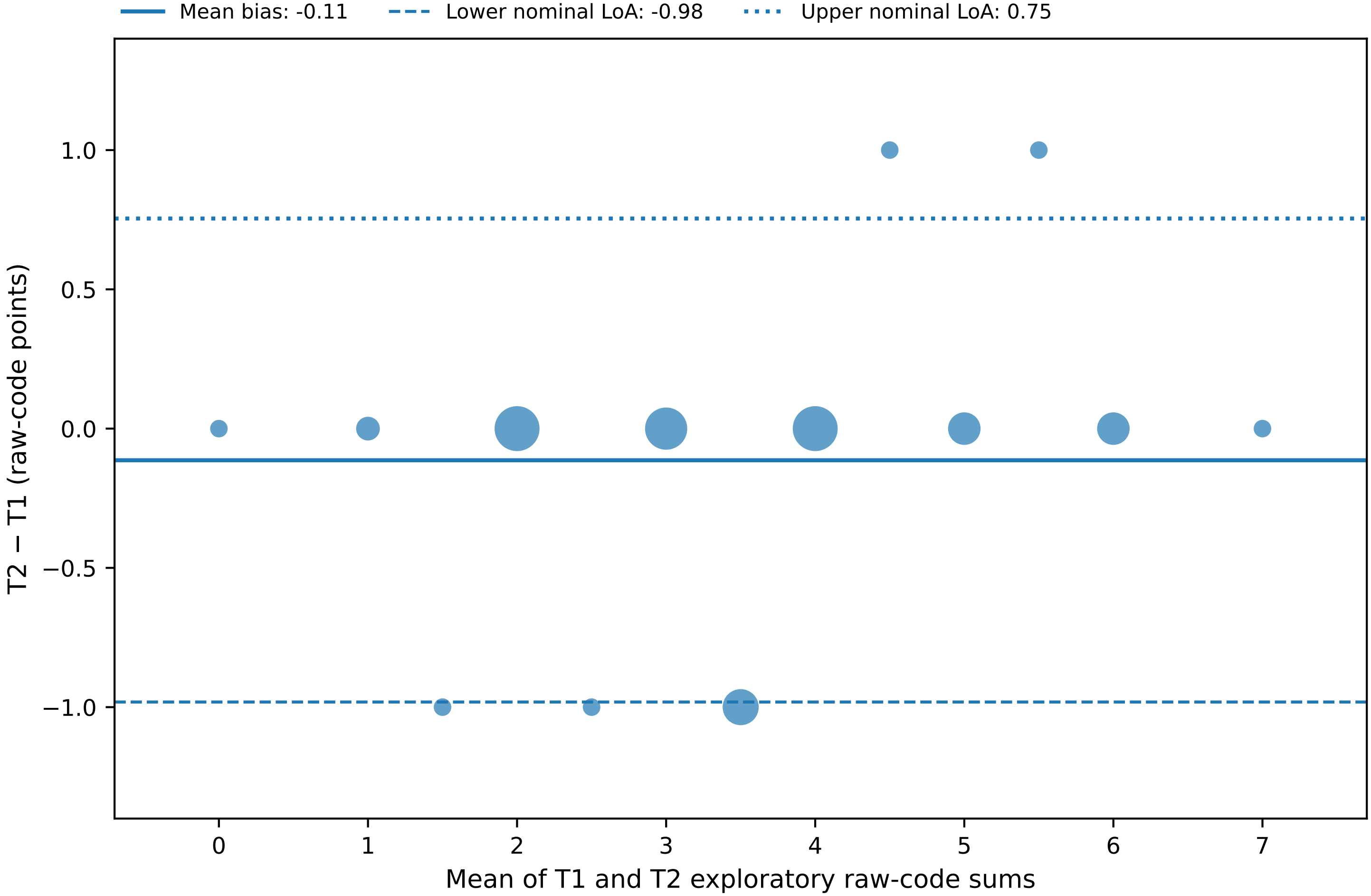

Each marker represents one or more observed pairs at identical coordinates; marker area is proportional to the number of pairs (maximum 8). Complete domain pairs were used, excluding non-applicable responses at either visit. Limits are mean difference ± 1.96 × SD of the differences. Discrete, zero-heavy differences mean that these normal-theory limits need not cover 95% of observed pairs. They are not validated clinical decision thresholds.

Figure S4. Domain 5: Making decisions

n = 44 paired observations | 3 items | no jitter or simulated observations

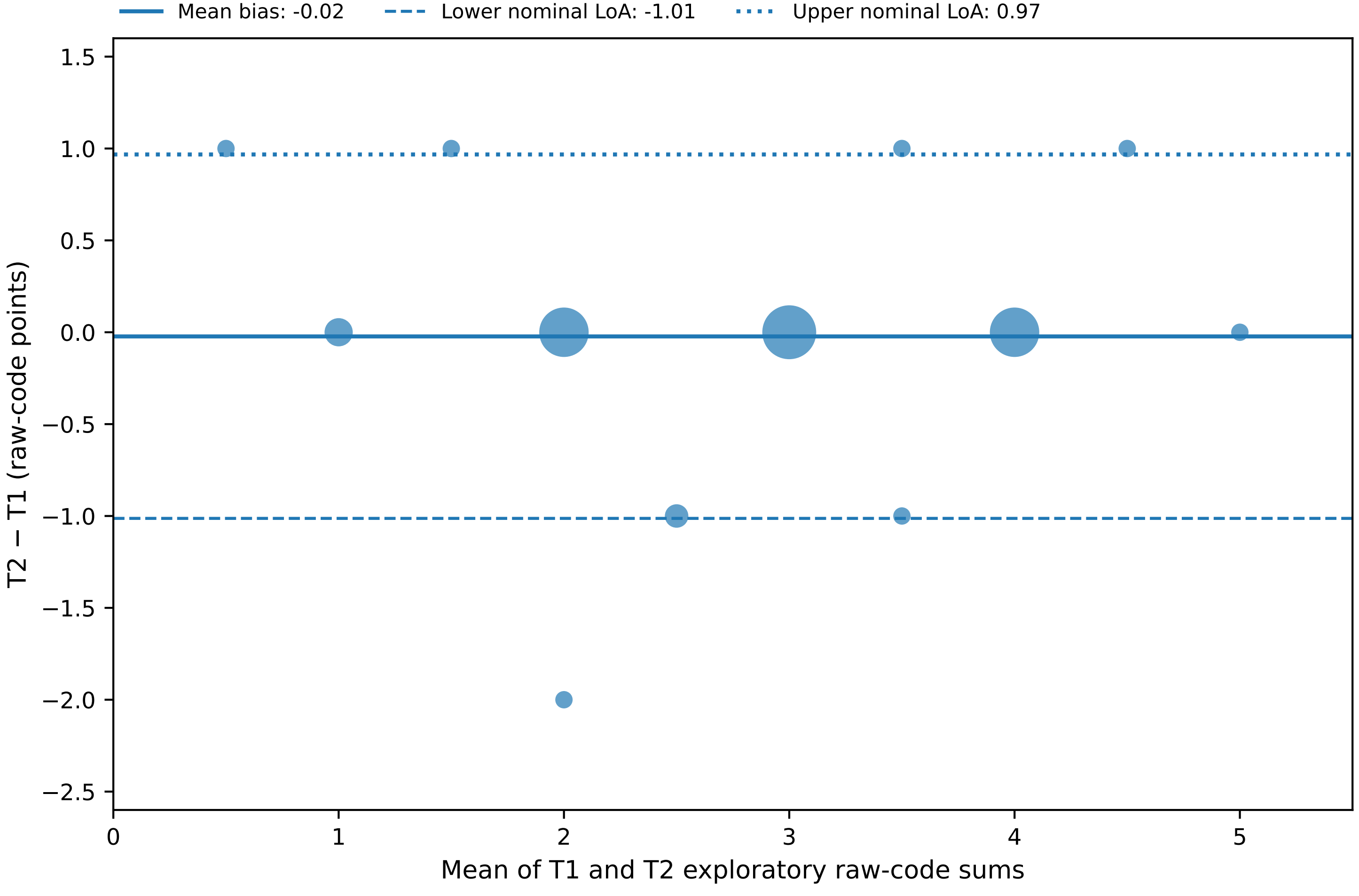

Each marker represents one or more observed pairs at identical coordinates; marker area is proportional to the number of pairs (maximum 12). Complete domain pairs were used, excluding non-applicable responses at either visit. Limits are mean difference ± 1.96 × SD of the differences. Discrete, zero-heavy differences mean that these normal-theory limits need not cover 95% of observed pairs. They are not validated clinical decision thresholds.

Figure S5. Domain 6: Undertaking a single task

n = 46 paired observations | 3 items | no jitter or simulated observations

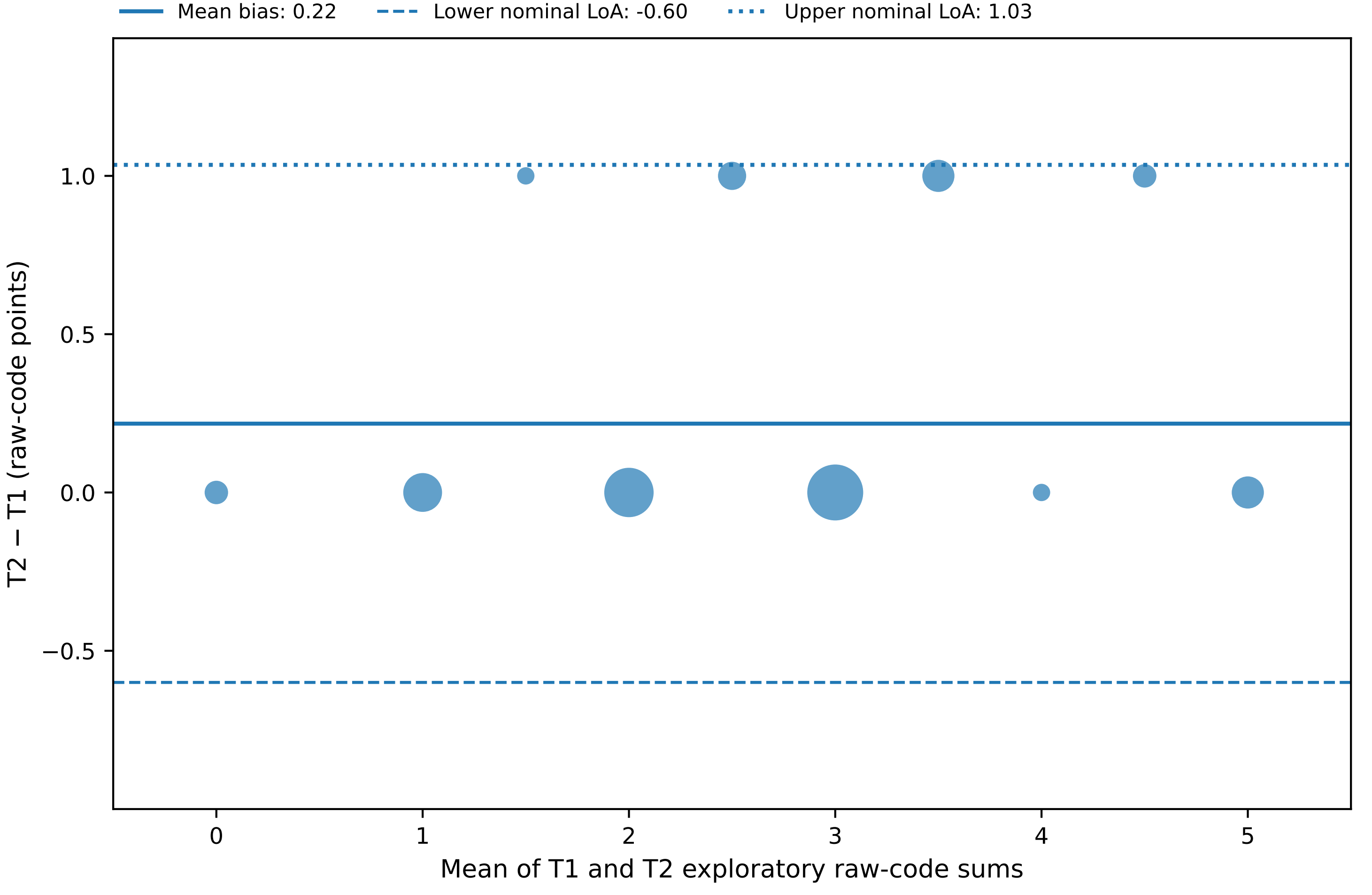

Each marker represents one or more observed pairs at identical coordinates; marker area is proportional to the number of pairs (maximum 13). Complete domain pairs were used, excluding non-applicable responses at either visit. Limits are mean difference ± 1.96 × SD of the differences. Discrete, zero-heavy differences mean that these normal-theory limits need not cover 95% of observed pairs. They are not validated clinical decision thresholds.

Figure S6. Domain 7: Managing one's own behaviour

n = 47 paired observations | 5 items | no jitter or simulated observations

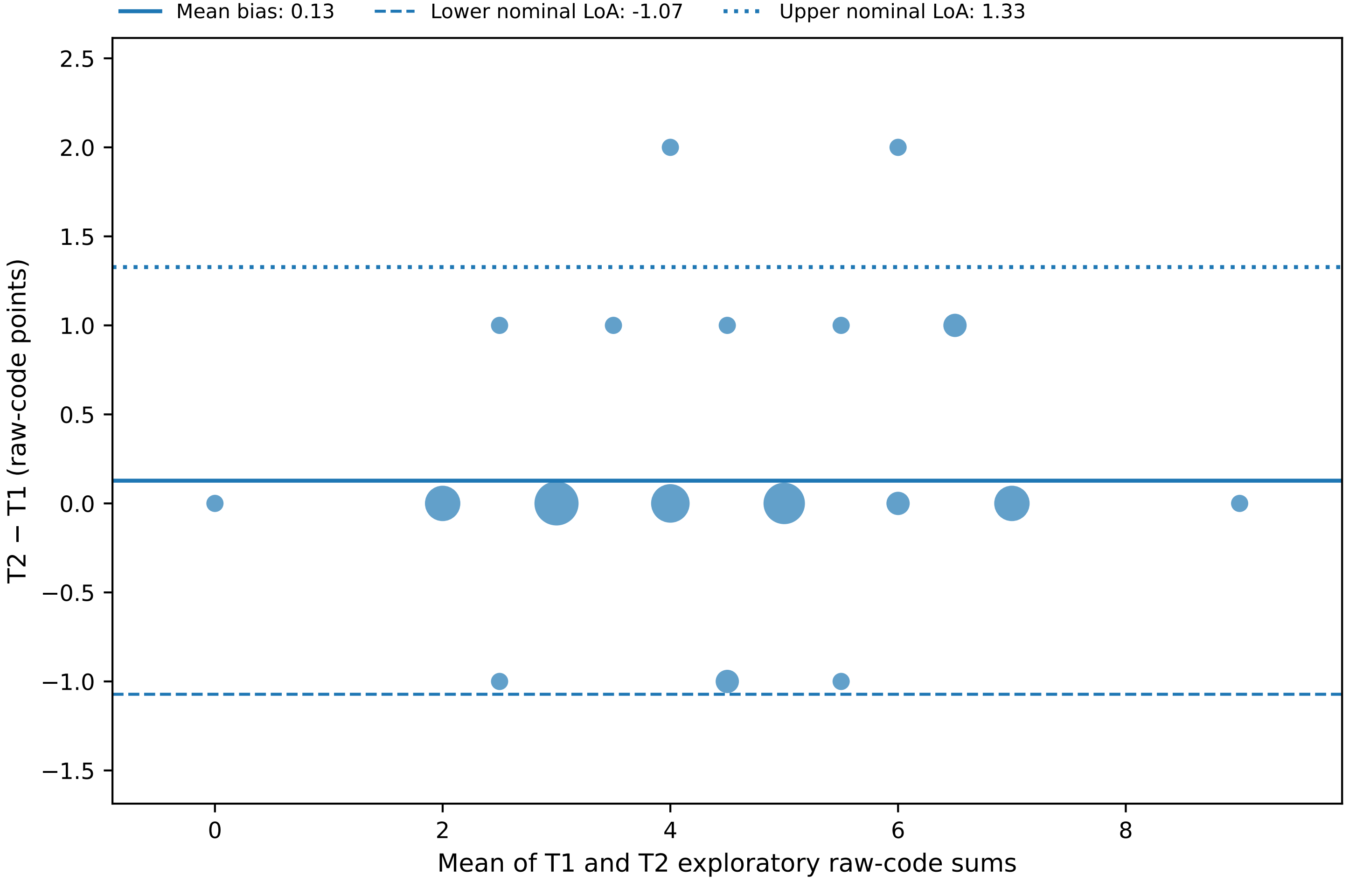

Each marker represents one or more observed pairs at identical coordinates; marker area is proportional to the number of pairs (maximum 8). Complete domain pairs were used, excluding non-applicable responses at either visit. Limits are mean difference ± 1.96 × SD of the differences. Discrete, zero-heavy differences mean that these normal-theory limits need not cover 95% of observed pairs. They are not validated clinical decision thresholds.

Figure S7. Domain 8: Conversation

n = 44 paired observations | 4 items | no jitter or simulated observations

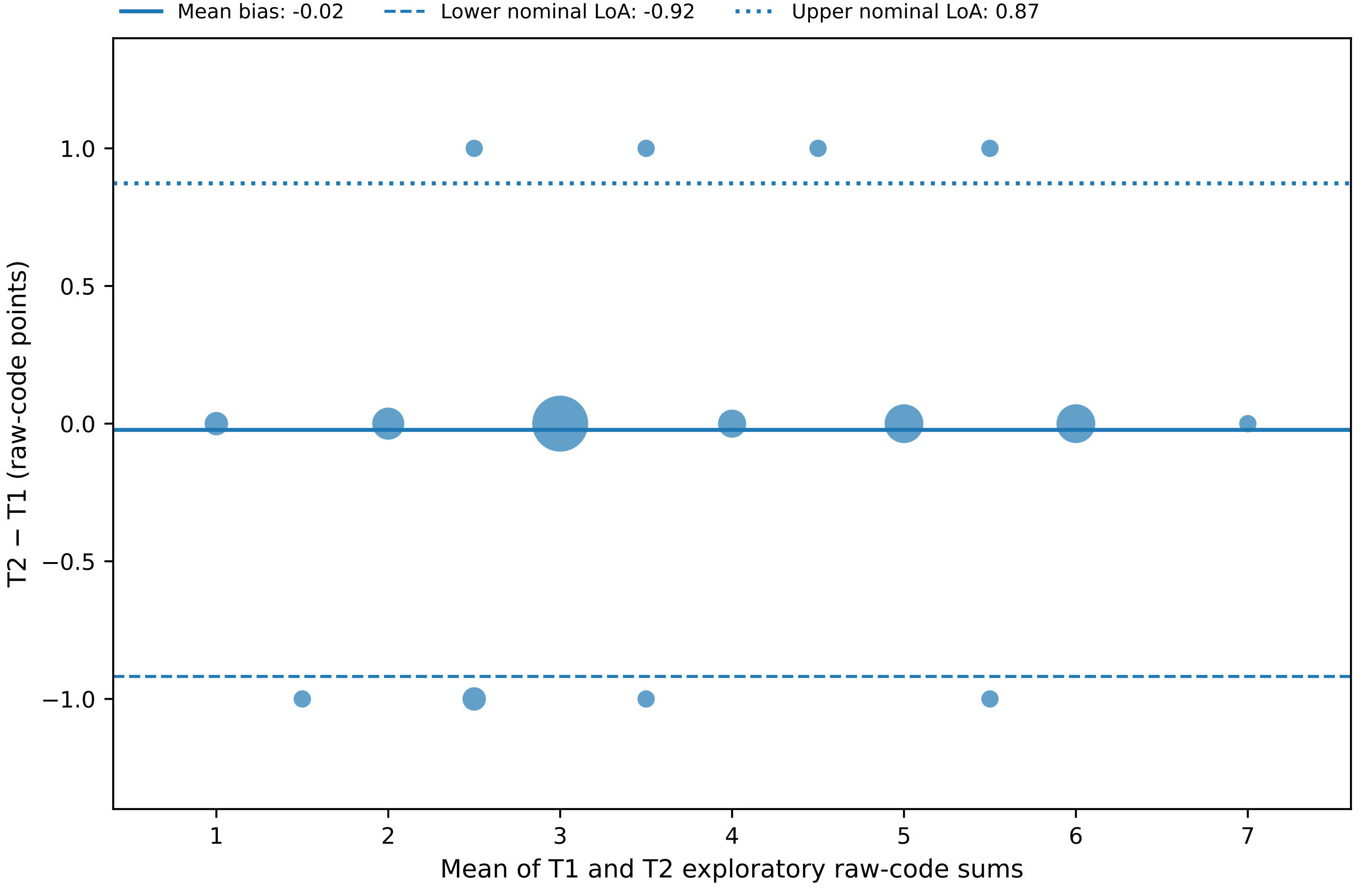

Each marker represents one or more observed pairs at identical coordinates; marker area is proportional to the number of pairs (maximum 13). Complete domain pairs were used, excluding non-applicable responses at either visit. Limits are mean difference ± 1.96 × SD of the differences. Discrete, zero-heavy differences mean that these normal-theory limits need not cover 95% of observed pairs. They are not validated clinical decision thresholds.

Figure S8. Domain 9: Changing basic body positions

n = 45 paired observations | 9 items | no jitter or simulated observations

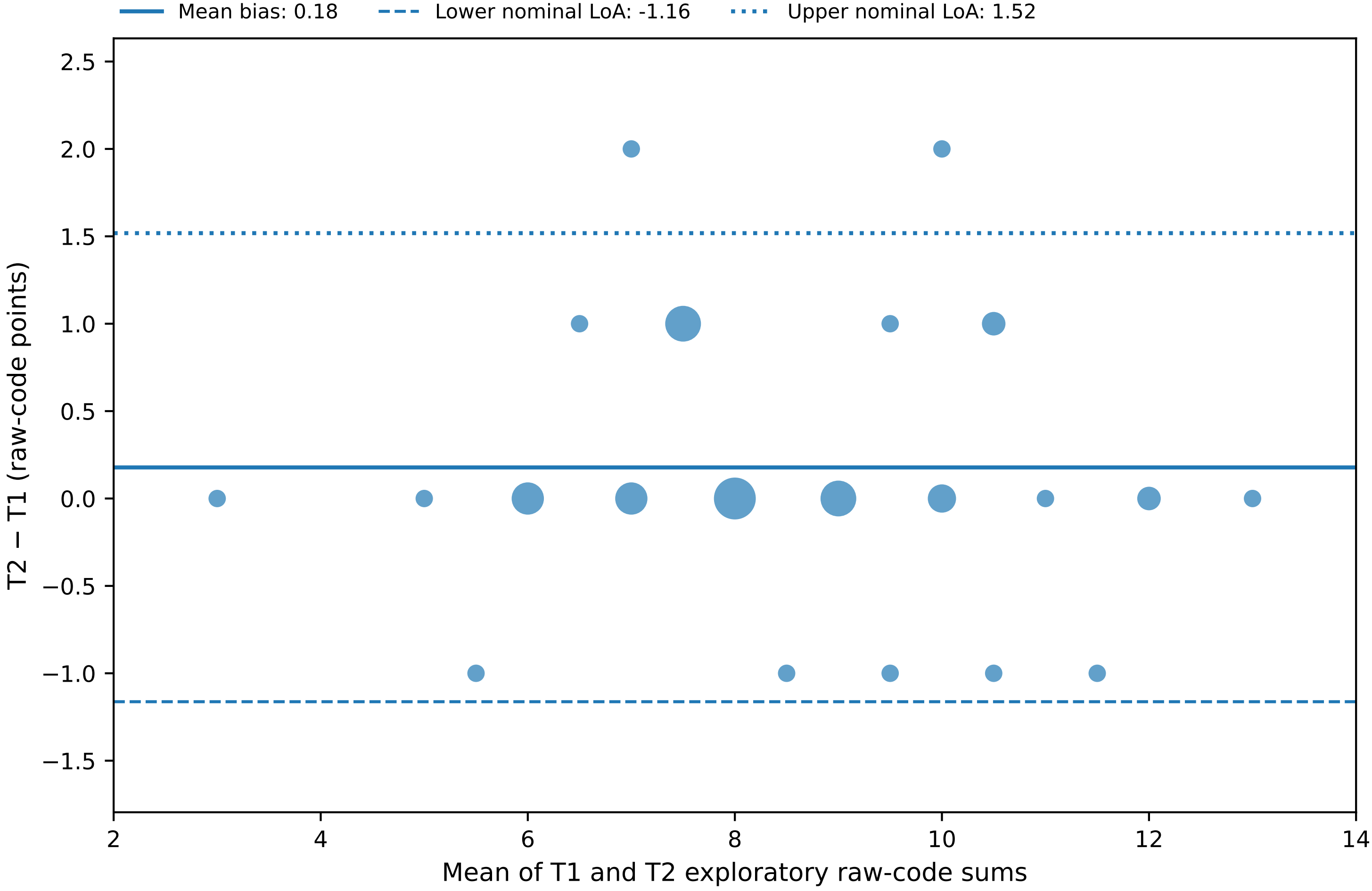

Each marker represents one or more observed pairs at identical coordinates; marker area is proportional to the number of pairs (maximum 7). Complete domain pairs were used, excluding non-applicable responses at either visit. Limits are mean difference ± 1.96 × SD of the differences. Discrete, zero-heavy differences mean that these normal-theory limits need not cover 95% of observed pairs. They are not validated clinical decision thresholds.

Figure S9. Domain 10: Maintaining a body position

n = 44 paired observations | 6 items | no jitter or simulated observations

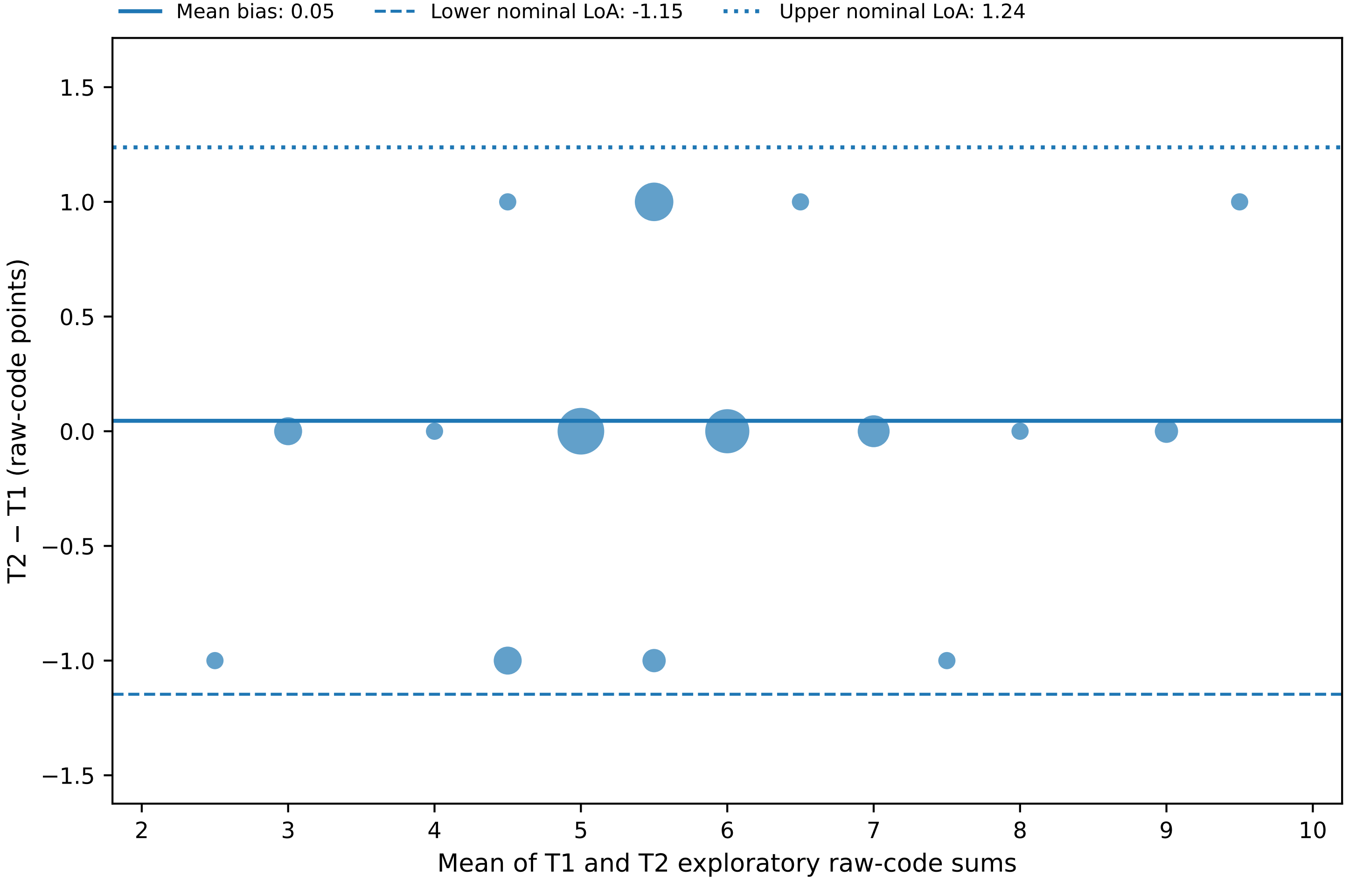

Each marker represents one or more observed pairs at identical coordinates; marker area is proportional to the number of pairs (maximum 9). Complete domain pairs were used, excluding non-applicable responses at either visit. Limits are mean difference ± 1.96 × SD of the differences. Discrete, zero-heavy differences mean that these normal-theory limits need not cover 95% of observed pairs. They are not validated clinical decision thresholds.

Figure S10. Domain 11: Hand and arm use

n = 44 paired observations | 4 items | no jitter or simulated observations

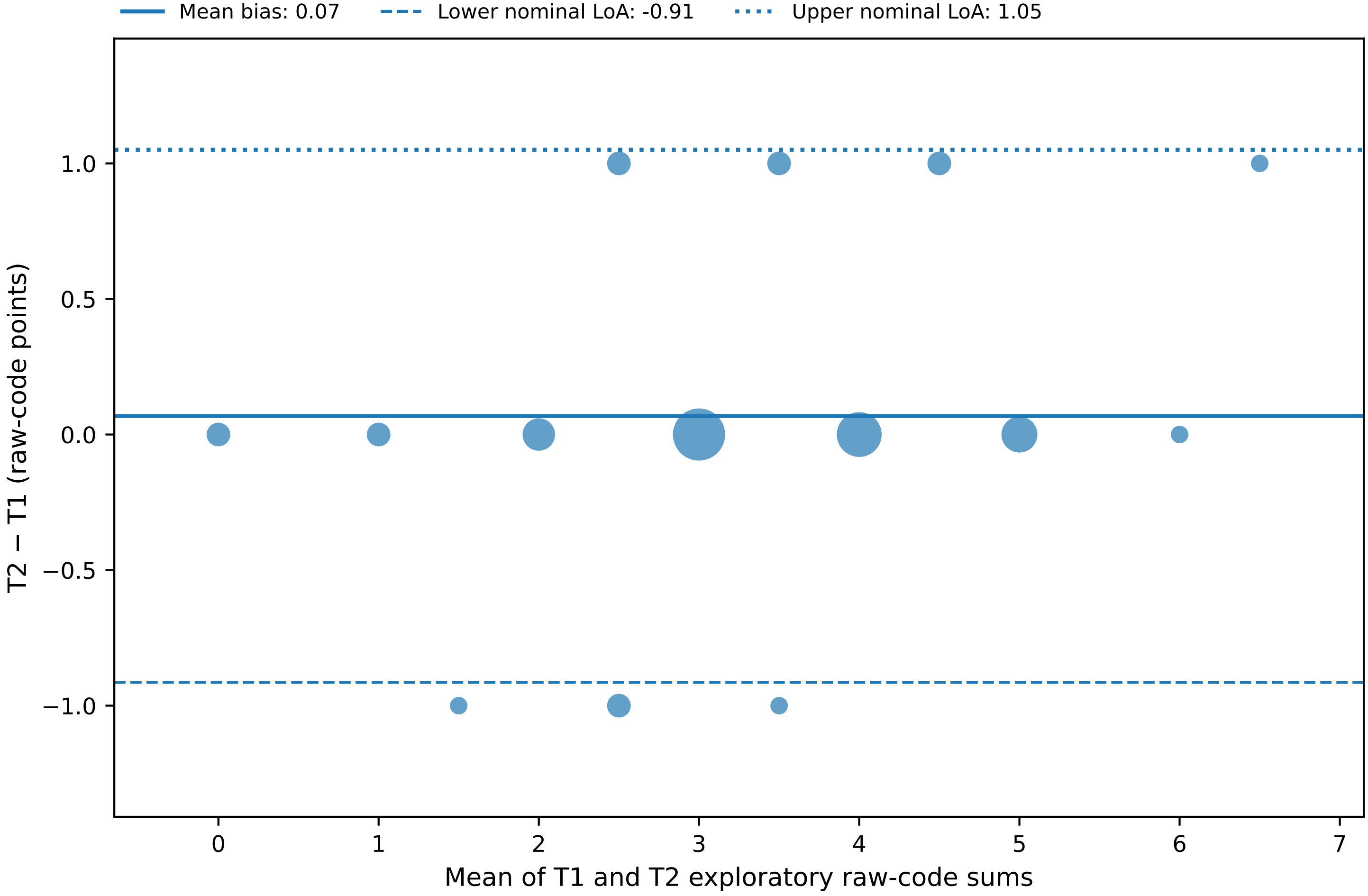

Each marker represents one or more observed pairs at identical coordinates; marker area is proportional to the number of pairs (maximum 11). Complete domain pairs were used, excluding non-applicable responses at either visit. Limits are mean difference ± 1.96 × SD of the differences. Discrete, zero-heavy differences mean that these normal-theory limits need not cover 95% of observed pairs. They are not validated clinical decision thresholds.

Figure S11. Domain 12: Walking

n = 43 paired observations | 8 items | no jitter or simulated observations

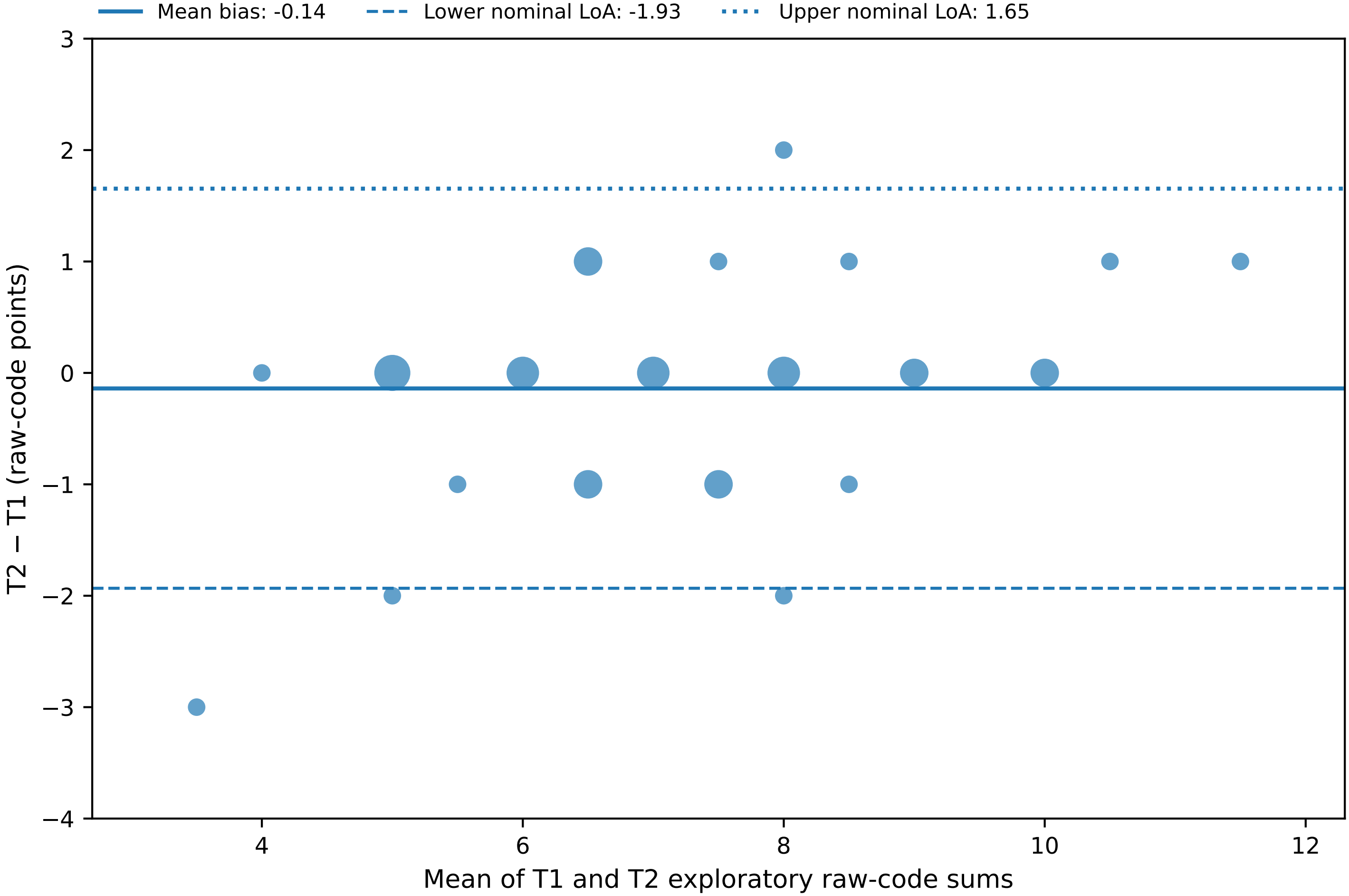

Each marker represents one or more observed pairs at identical coordinates; marker area is proportional to the number of pairs (maximum 5). Complete domain pairs were used, excluding non-applicable responses at either visit. Limits are mean difference ± 1.96 × SD of the differences. Discrete, zero-heavy differences mean that these normal-theory limits need not cover 95% of observed pairs. They are not validated clinical decision thresholds.

Figure S12. Domain 13: Moving around [available-item subset]

n = 47 paired observations | 5 items | no jitter or simulated observations

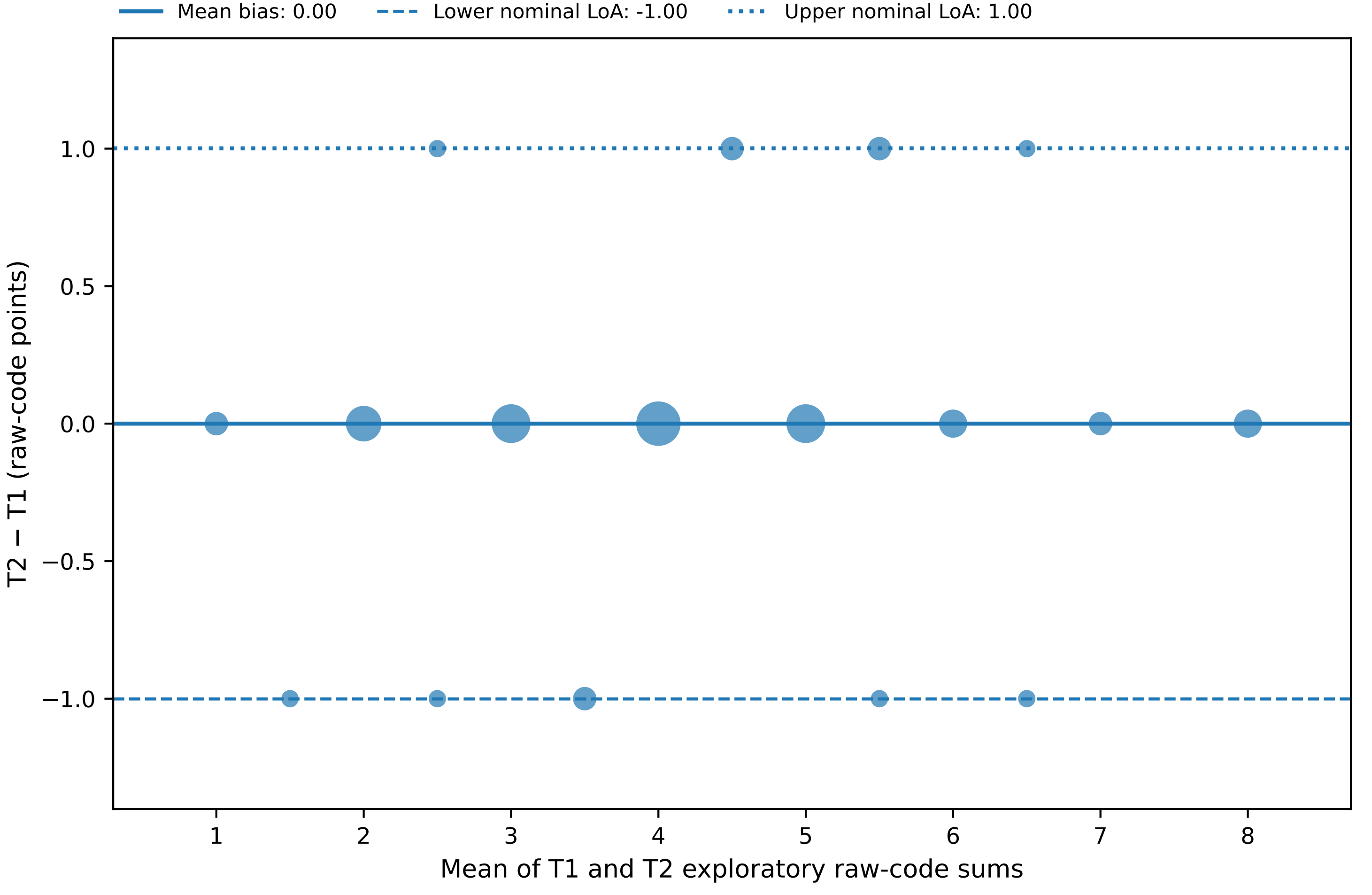

Each marker represents one or more observed pairs at identical coordinates; marker area is proportional to the number of pairs (maximum 8). Complete domain pairs were used, excluding non-applicable responses at either visit. Limits are mean difference ± 1.96 × SD of the differences. Discrete, zero-heavy differences mean that these normal-theory limits need not cover 95% of observed pairs. They are not validated clinical decision thresholds.

Figure S13. Domain 14: Moving around in different locations

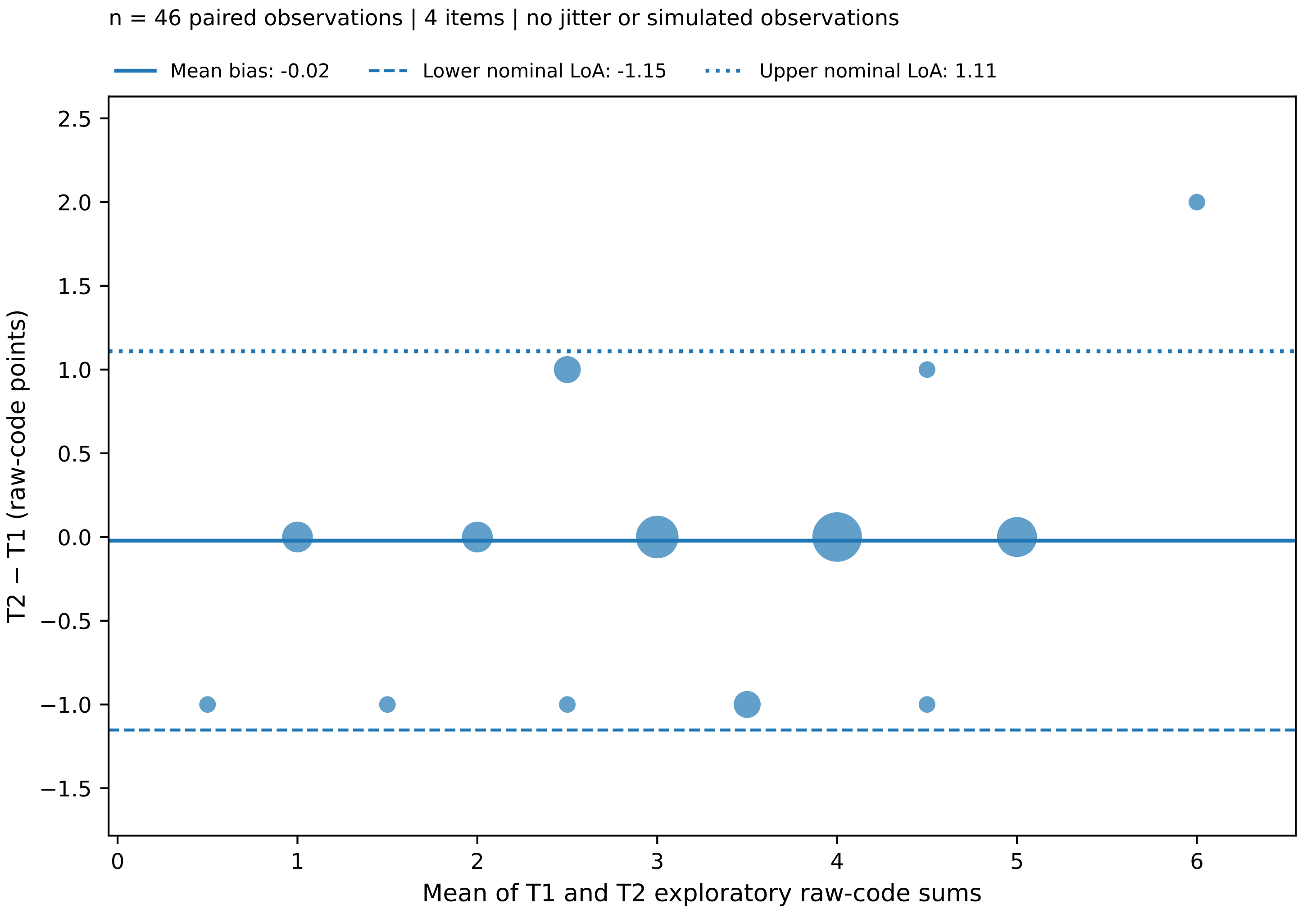

Each marker represents one or more observed pairs at identical coordinates; marker area is proportional to the number of pairs (maximum 11). Complete domain pairs were used, excluding non-applicable responses at either visit. Limits are mean difference ± 1.96 × SD of the differences. Discrete, zero-heavy differences mean that these normal-theory limits need not cover 95% of observed pairs. They are not validated clinical decision thresholds.

Figure S14. Domain 15: Caring for body parts

n = 43 paired observations | 5 items | no jitter or simulated observations

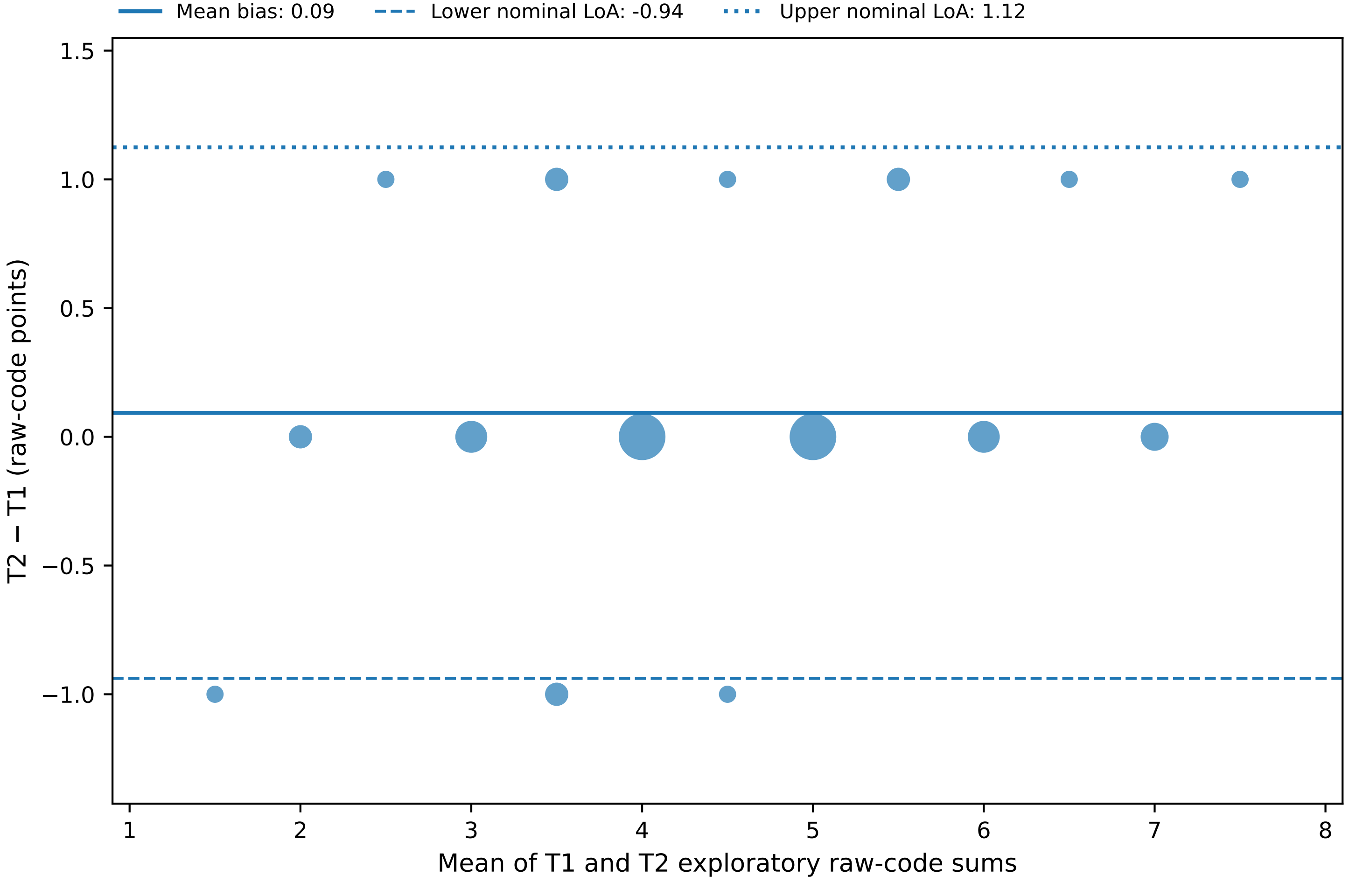

Each marker represents one or more observed pairs at identical coordinates; marker area is proportional to the number of pairs (maximum 9). Complete domain pairs were used, excluding non-applicable responses at either visit. Limits are mean difference ± 1.96 × SD of the differences. Discrete, zero-heavy differences mean that these normal-theory limits need not cover 95% of observed pairs. They are not validated clinical decision thresholds.

Figure S15. Domain 16: Dressing

n = 46 paired observations | 8 items | no jitter or simulated observations

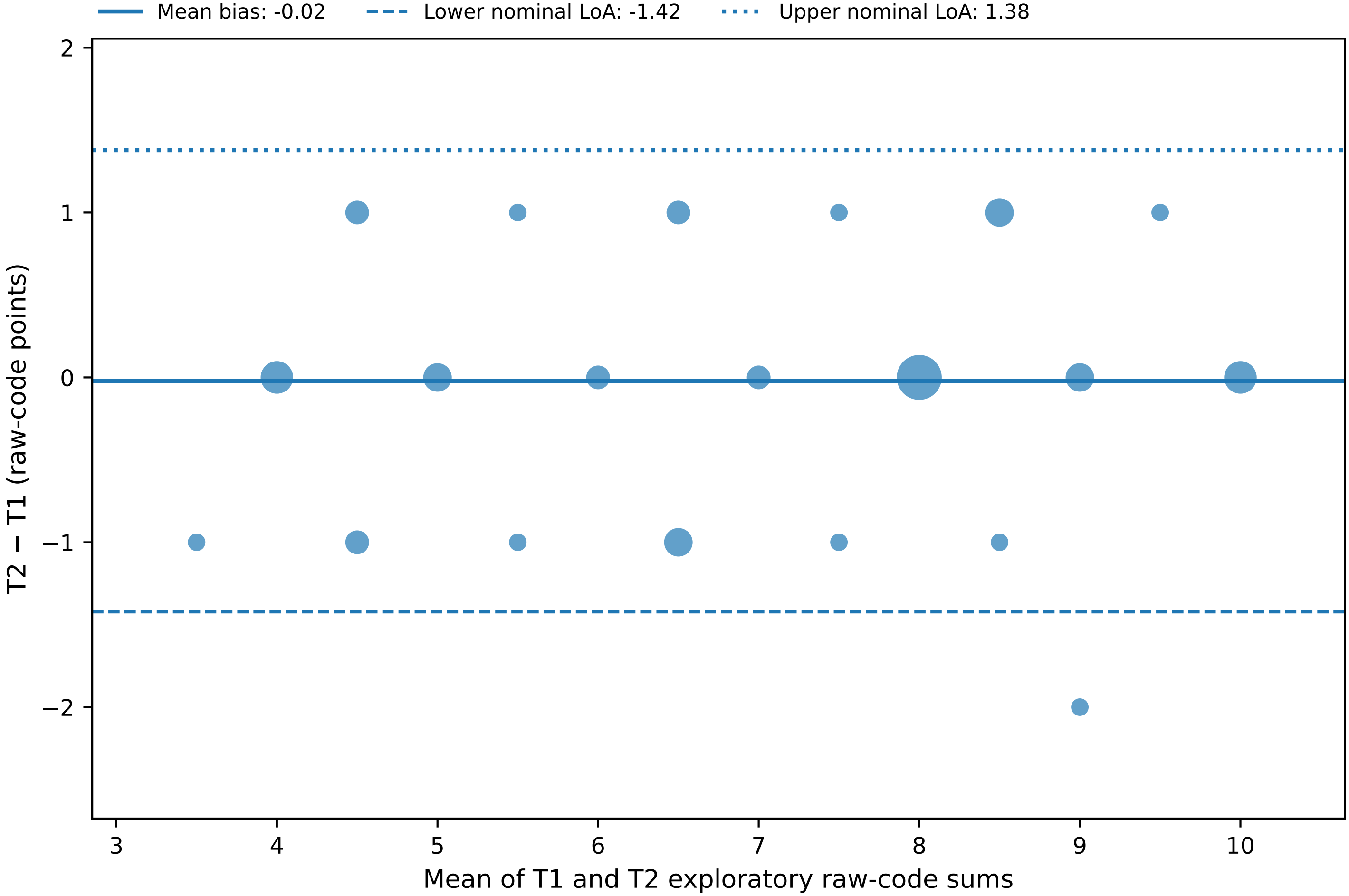

Each marker represents one or more observed pairs at identical coordinates; marker area is proportional to the number of pairs (maximum 8). Complete domain pairs were used, excluding non-applicable responses at either visit. Limits are mean difference ± 1.96 × SD of the differences. Discrete, zero-heavy differences mean that these normal-theory limits need not cover 95% of observed pairs. They are not validated clinical decision thresholds.

Figure S16. Domain 17: Basic interpersonal interactions

n = 45 paired observations | 4 items | no jitter or simulated observations

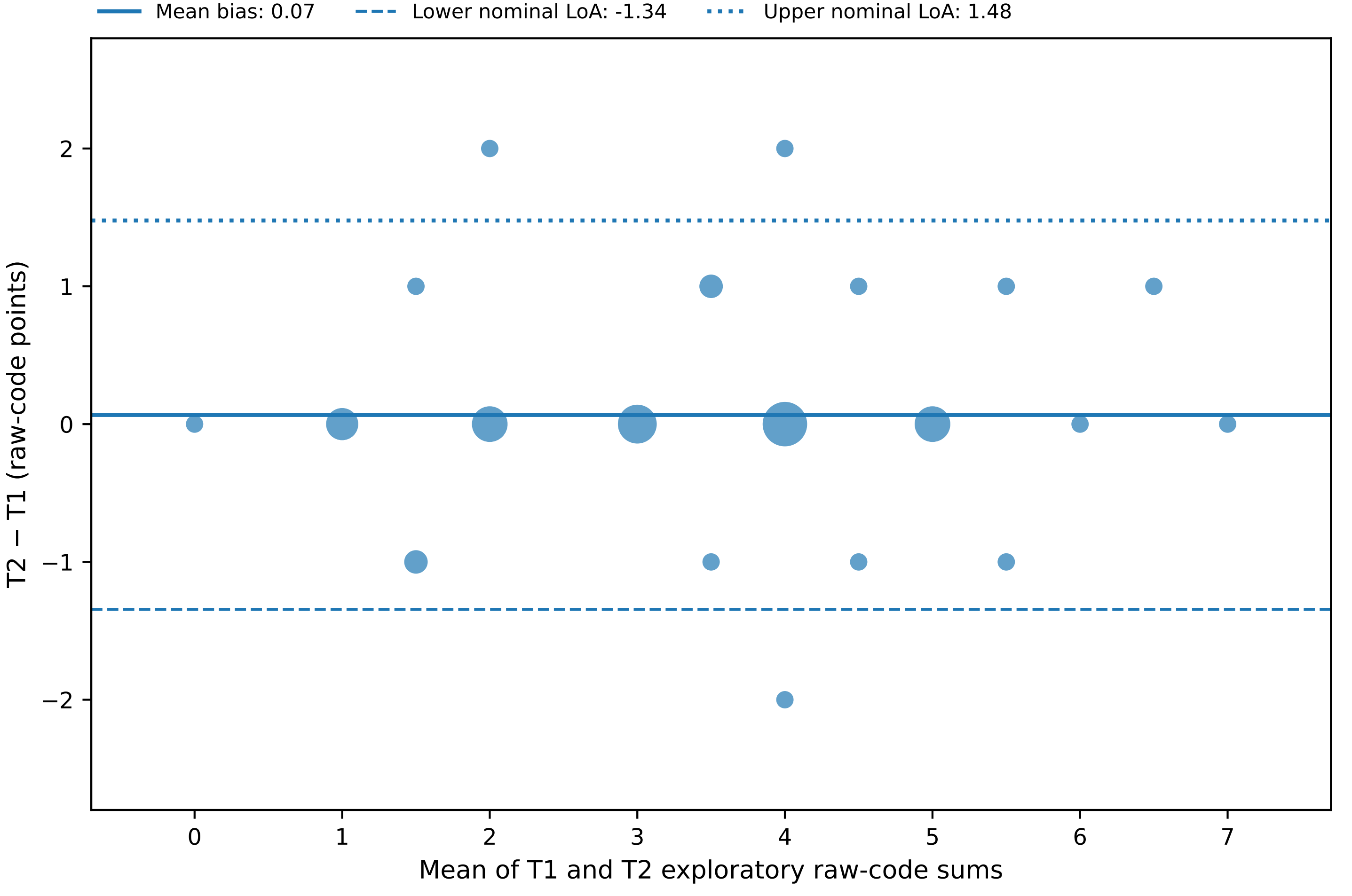

Each marker represents one or more observed pairs at identical coordinates; marker area is proportional to the number of pairs (maximum 8). Complete domain pairs were used, excluding non-applicable responses at either visit. Limits are mean difference ± 1.96 × SD of the differences. Discrete, zero-heavy differences mean that these normal-theory limits need not cover 95% of observed pairs. They are not validated clinical decision thresholds.

Figure S17. Domain 18: Informal relationships with peers

n = 47 paired observations | 3 items | no jitter or simulated observations

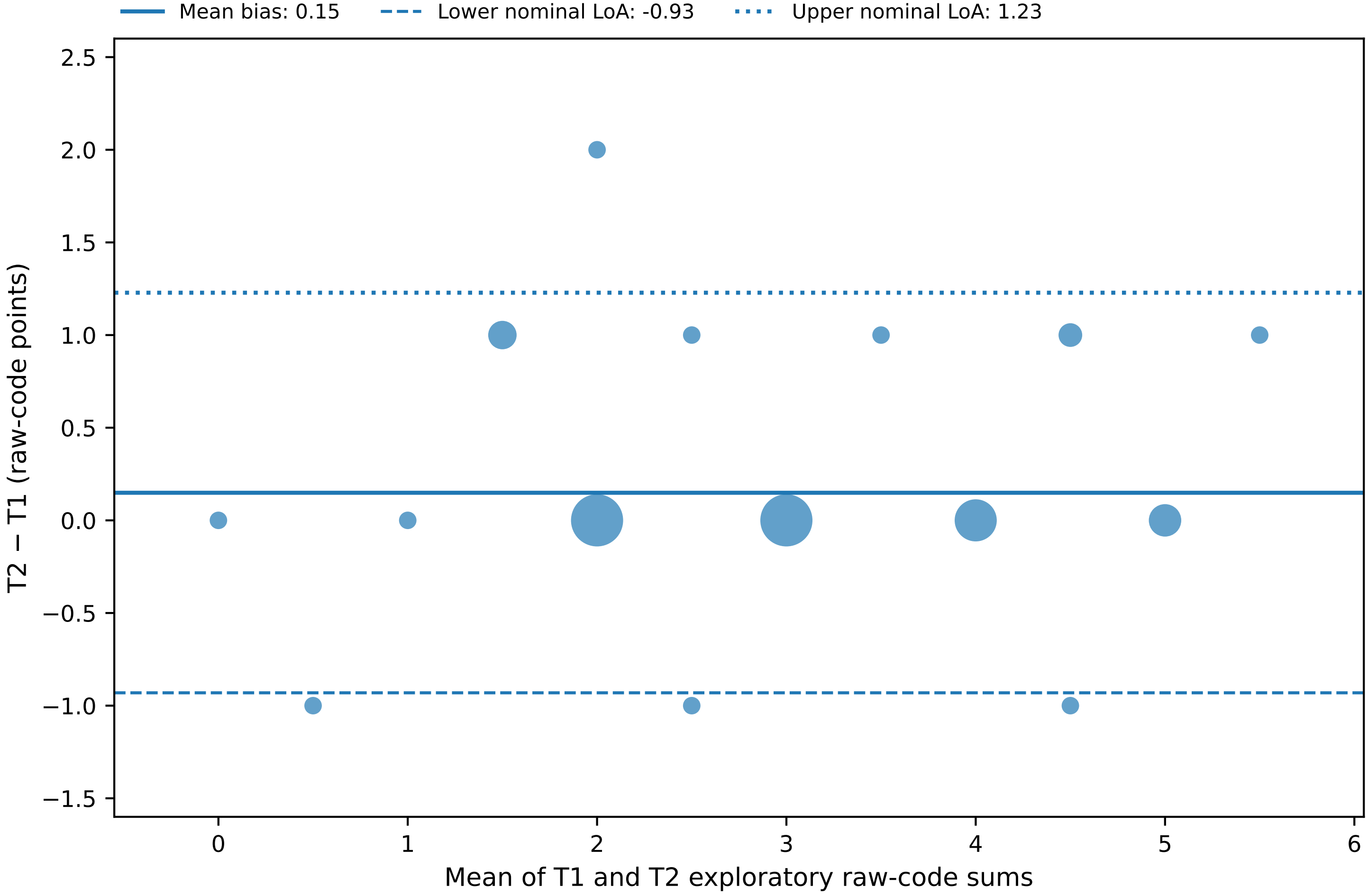

Each marker represents one or more observed pairs at identical coordinates; marker area is proportional to the number of pairs (maximum 11). Complete domain pairs were used, excluding non-applicable responses at either visit. Limits are mean difference ± 1.96 × SD of the differences. Discrete, zero-heavy differences mean that these normal-theory limits need not cover 95% of observed pairs. They are not validated clinical decision thresholds.

Figure S18. Domain 19: Family relationships

n = 46 paired observations | 4 items | no jitter or simulated observations

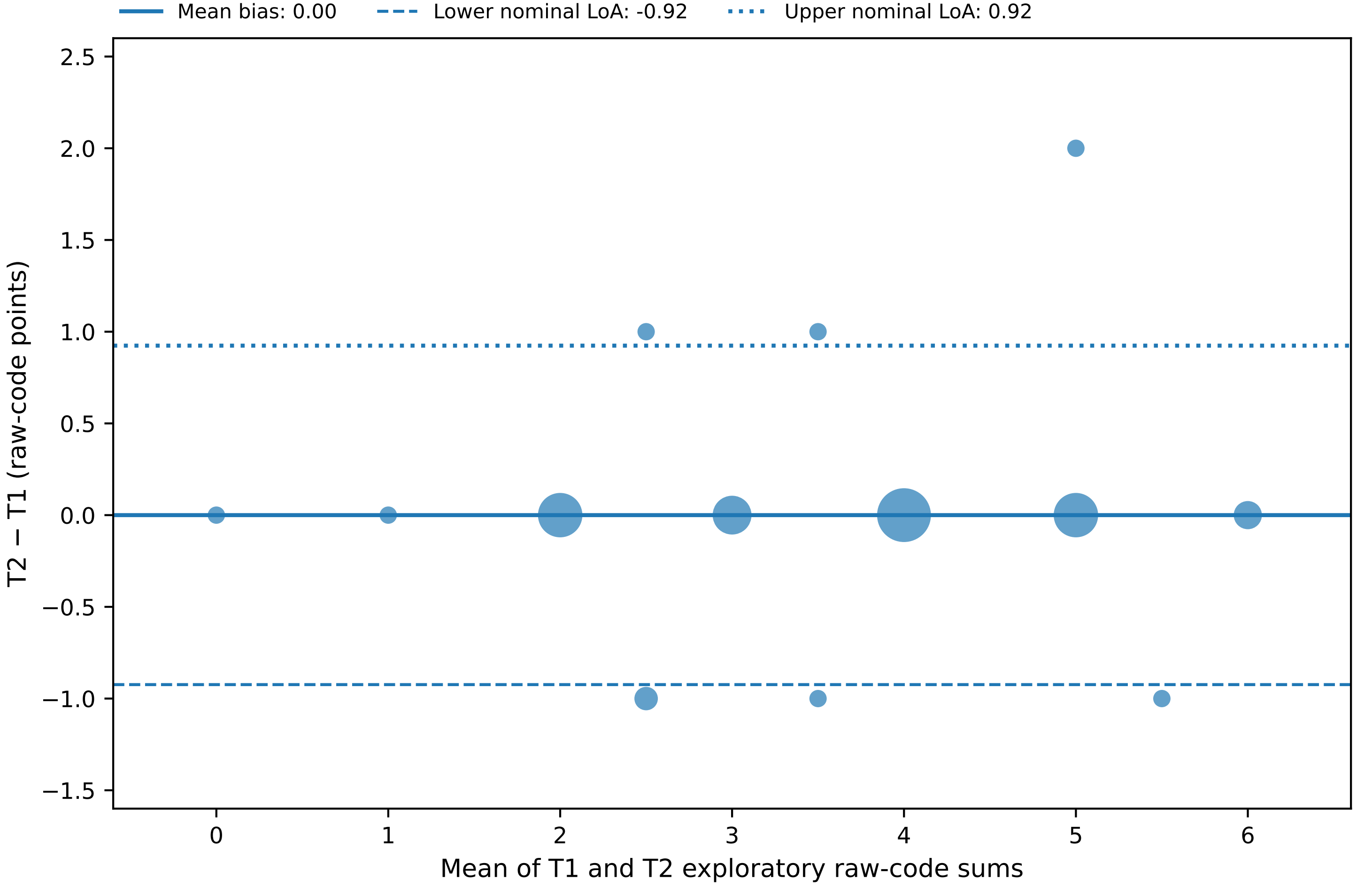

Each marker represents one or more observed pairs at identical coordinates; marker area is proportional to the number of pairs (maximum 12). Complete domain pairs were used, excluding non-applicable responses at either visit. Limits are mean difference ± 1.96 × SD of the differences. Discrete, zero-heavy differences mean that these normal-theory limits need not cover 95% of observed pairs. They are not validated clinical decision thresholds.

Figure S19. Domain 20: Engagement in play

n = 45 paired observations | 5 items | no jitter or simulated observations

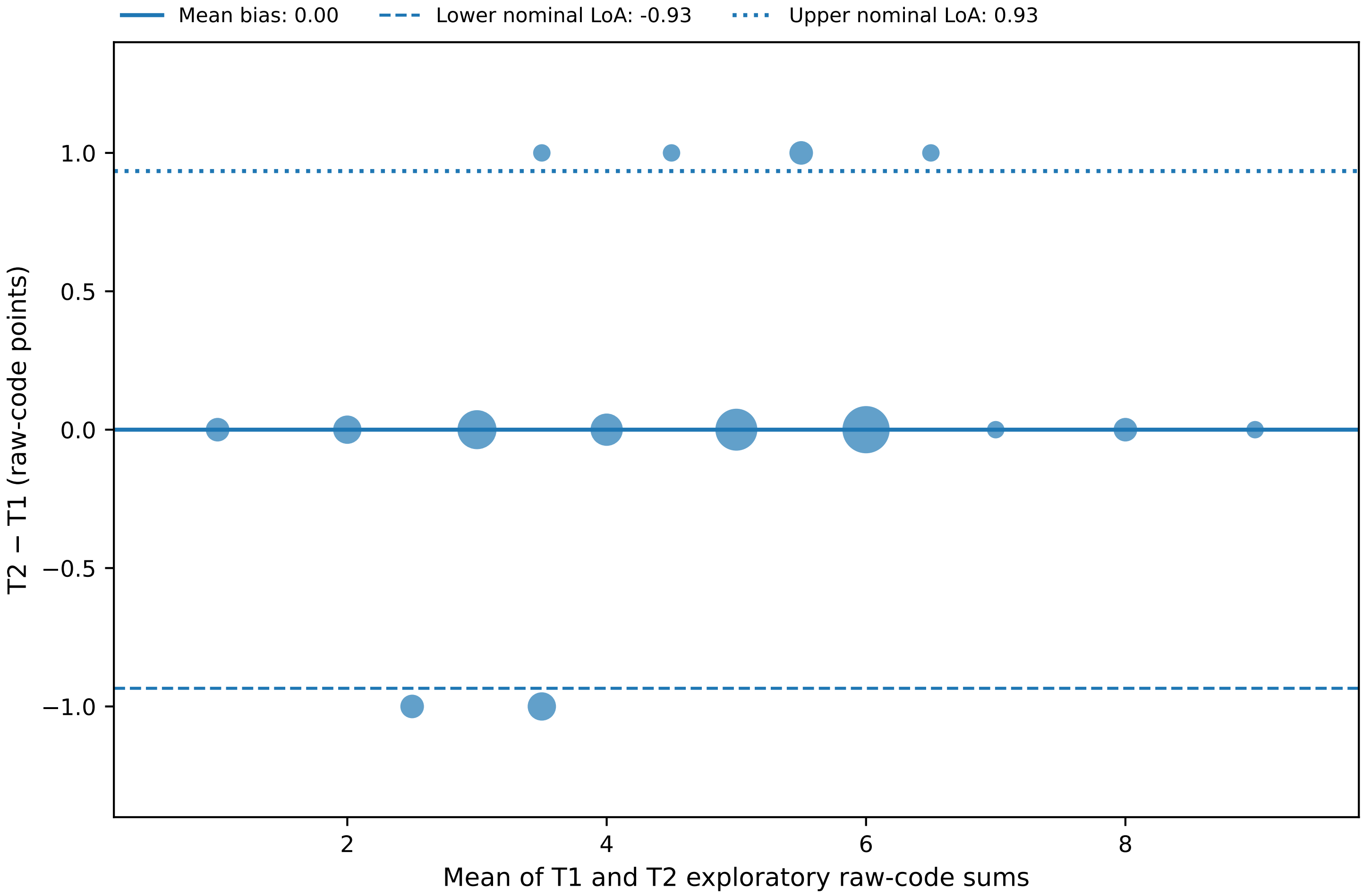

Each marker represents one or more observed pairs at identical coordinates; marker area is proportional to the number of pairs (maximum 9). Complete domain pairs were used, excluding non-applicable responses at either visit. Limits are mean difference ± 1.96 × SD of the differences. Discrete, zero-heavy differences mean that these normal-theory limits need not cover 95% of observed pairs. They are not validated clinical decision thresholds.

Figure S20. Domain 21: Recreation and leisure [available-item subset]

n = 43 paired observations | 6 items | no jitter or simulated observations

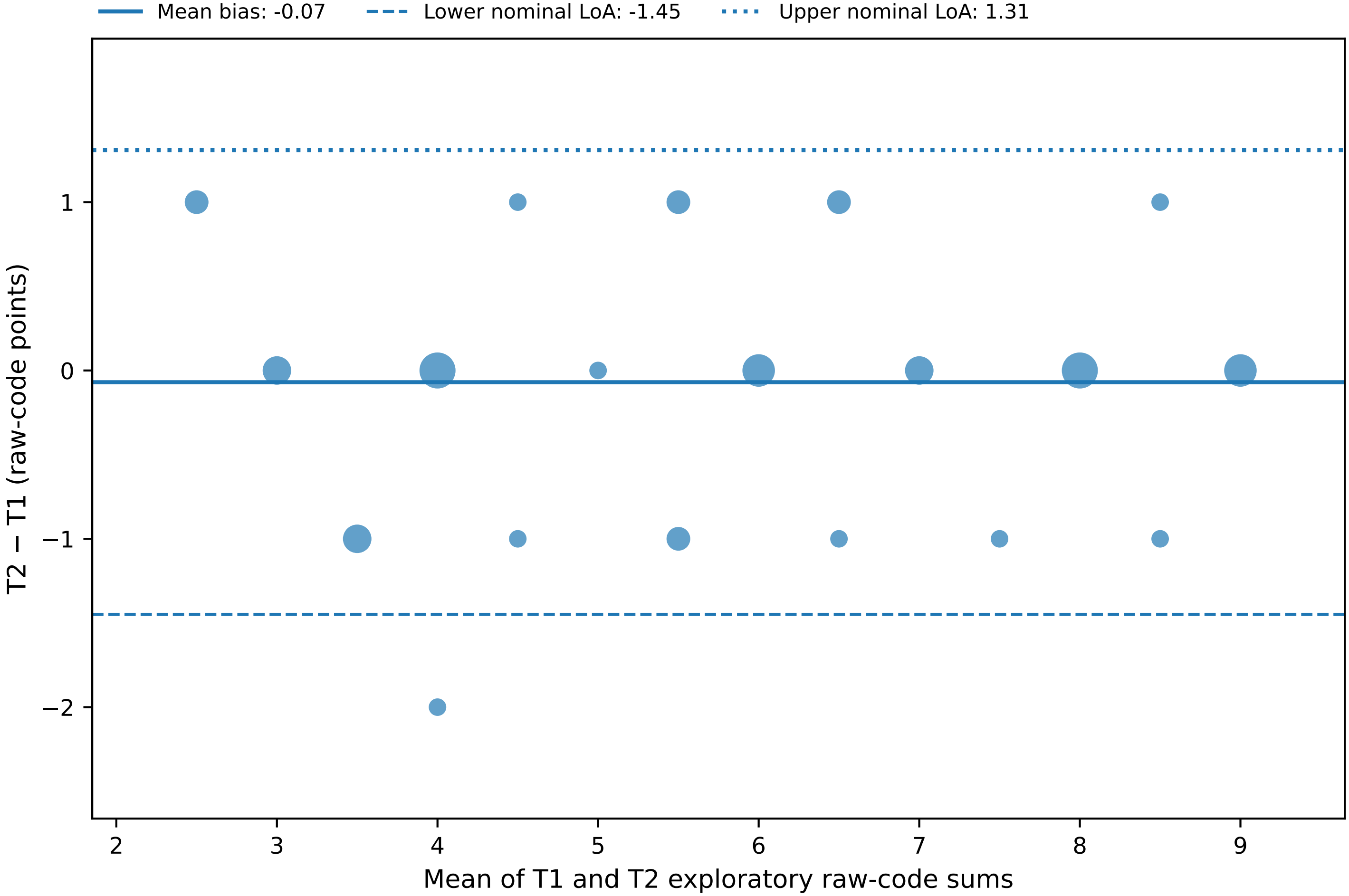

Each marker represents one or more observed pairs at identical coordinates; marker area is proportional to the number of pairs (maximum 5). Complete domain pairs were used, excluding non-applicable responses at either visit. Limits are mean difference ± 1.96 × SD of the differences. Discrete, zero-heavy differences mean that these normal-theory limits need not cover 95% of observed pairs. They are not validated clinical decision thresholds.
