# Supplementary material for: Greek Translation and Preliminary Test–Retest Reliability of the AQUA CHILD Questionnaire in Children With Developmental Disabilities
Source: Physiother Res Int. 2026 Oct 8;31(4):e70357. doi: 10.1002/pri.70357 (PMC13648008; doi:10.1002/pri.70357)
Supplement: Supplementary file 2 [file PRI-31-e70357-s001.docx]

# Table S2. Item-to-domain crosswalk

Item identifiers follow the form domain and within-domain number. Descriptors identify the source content and are not new item wording. “Absent” means the variable is not present in either CSV; it does not mean a child was incapable or the item was non-applicable.

| **Domain** | **Item ID** | **Content descriptor from the supplied English form** | **Electronic data** |
| --- | --- | --- | --- |
| 1 | 1.1 | Backstroke | Available |
| 1 | 1.2 | Crawl stroke | Available |
| 1 | 1.3 | Breaststroke | Available |
| 1 | 1.4 | Another stroke | Available |
| 2 | 2.1 | Use new utensils for food | Available |
| 2 | 2.2 | Use work tools/tools from home for purposes other than their original purpose | Available |
| 2 | 2.3 | Develop a new game with games or toys around | Available |
| 3 | 3.1 | Focusing attention on the person in contact with him/her - on his/her instructions or requests | Available |
| 3 | 3.2 | Focusing on the task while ignoring the distracting stimuli in the environment | Available |
| 4 | 4.1 | Identify what the problem is and define it | Available |
| 4 | 4.2 | Propose possible solutions and ways to solve it | Available |
| 4 | 4.3 | Discuss with others and evaluate the possible impact of the solutions | Available |
| 4 | 4.4 | Execute the chosen solution | Available |
| 5 | 5.1 | Choose a certain item/game to perform the required action | Available |
| 5 | 5.2 | Decide from among several similar options | Available |
| 5 | 5.3 | Perform one task from among several tasks that were raised as possible | Available |
| 6 | 6.1 | Initiate the task (alone/in a group) | Available |
| 6 | 6.2 | Organize what is needed - materials or a place to do the task (alone/in a group) | Available |
| 6 | 6.3 | Complete the task (alone/in a group) | Available |
| 7 | 7.1 | Stay in a new environment | Available |
| 7 | 7.2 | Meet new classroom requirements | Available |
| 7 | 7.3 | Get close to new people | Available |
| 7 | 7.4 | Adjust the level of activity to the situation (for example, be quiet in the classroom when required) | Available |
| 7 | 7.5 | Change from one state to another - like returning from a break to class | Available |
| 8 | 8.1 | Spoken language | Available |
| 8 | 8.2 | Written language | Available |
| 8 | 8.3 | Sign language | Available |
| 8 | 8.4 | Another form of conversation | Available |
| 9 | 9.1 | Lying down and rolling from side to side | Available |
| 9 | 9.2 | Transition from lying down to sitting | Available |
| 9 | 9.3 | Transition from sitting to lying down | Available |
| 9 | 9.4 | Transition from sitting on a chair/bed to standing | Available |
| 9 | 9.5 | Transition from standing to sitting on a chair/bed | Available |
| 9 | 9.6 | Transition from standing to sitting on the floor | Available |
| 9 | 9.7 | Transition from sitting on the floor to standing | Available |
| 9 | 9.8 | Entering and exiting positions of kneeling or sitting bent over | Available |
| 9 | 9.9 | Bending and moving the body's center of gravity | Available |
| 10 | 10.1 | Maintaining the head position | Available |
| 10 | 10.2 | Maintaining a supine position | Available |
| 10 | 10.3 | Maintaining a squatting position | Available |
| 10 | 10.4 | Maintaining a kneeling position | Available |
| 10 | 10.5 | Maintaining a sitting position | Available |
| 10 | 10.6 | Maintaining a standing position | Available |
| 11 | 11.1 | Turning/pressing door handles to open them | Available |
| 11 | 11.2 | Throwing or catching an object | Available |
| 11 | 11.3 | Pulling or pushing objects | Available |
| 11 | 11.4 | Reaching out to a person or object | Available |
| 12 | 12.1 | walk forward - 5 steps | Available |
| 12 | 12.2 | walking backward - 5 steps | Available |
| 12 | 12.3 | walking sideways - 5 steps to the right side | Available |
| 12 | 12.4 | walking sideways - 5 steps to the left side | Available |
| 12 | 12.5 | Walking a short distance - up to 10 meters | Available |
| 12 | 12.6 | Walking a distance of over 10 meters | Available |
| 12 | 12.7 | Walking on different surfaces - sand, grass, pavement | Available |
| 12 | 12.8 | Walking around obstacles - at home, outside | Available |
| 13 | 13.1 | Running/jogging at home or outside | Available |
| 13 | 13.2 | Jumping | Available |
| 13 | 13.3 | Crawling | Available |
| 13 | 13.4 | Climbing obstacles | Available |
| 13 | 13.5 | Mobility on a manual wheelchair | Available |
| 13 | 13.6 | Mobility on an electric wheelchair/scooter or other electric vehicle | ABSENT |
| 14 | 14.1 | Moving between rooms in the house | Available |
| 14 | 14.2 | Moving inside other buildings, except the house | Available |
| 14 | 14.3 | Moving around in the school - classroom and yard | Available |
| 14 | 14.4 | Moving around on the streets in the city | Available |
| 15 | 15.1 | Care for skin – applying cream or bandage | Available |
| 15 | 15.2 | Brush teeth | Available |
| 15 | 15.3 | Comb and gather the hair | Available |
| 15 | 15.4 | Blow the nose | Available |
| 15 | 15.5 | Cut nails | Available |
| 16 | 16.1 | Shirts, blouses | Available |
| 16 | 16.2 | Pants, skirts | Available |
| 16 | 16.3 | Underwear, tights | Available |
| 16 | 16.4 | Hats | Available |
| 16 | 16.5 | Gloves | Available |
| 16 | 16.6 | Coats | Available |
| 16 | 16.7 | Shoes, boots, sandals and slippers | Available |
| 16 | 16.8 | Splints/ prostheses | Available |
| 17 | 17.1 | Demonstrating consideration and appreciation for another | Available |
| 17 | 17.2 | Appropriate response to the feelings of others | Available |
| 17 | 17.3 | Demonstration of respect, warmth and tolerance | Available |
| 17 | 17.4 | Use of appropriate physical contact | Available |
| 18 | 18.1 | Children from the class | Available |
| 18 | 18.2 | Children from the sport and leisure activities | Available |
| 18 | 18.3 | Children from the neighborhood | Available |
| 19 | 19.1 | You, the parents | Available |
| 19 | 19.2 | Brothers | Available |
| 19 | 19.3 | Grandparents | Available |
| 19 | 19.4 | Uncles, aunts and cousins | Available |
| 20 | 20.1 | Game alone with the games around | Available |
| 20 | 20.2 | Watching the game of others with interest (observation) | Available |
| 20 | 20.3 | Playing at the same time as others without actual cooperation | Available |
| 20 | 20.4 | Playing with others in pair cooperation | Available |
| 20 | 20.5 | Playing with others in cooperation in a group over 2 children | Available |
| 21 | 21.1 | Participation in physical activity | Available |
| 21 | 21.2 | Engaging in art or creative hobbies | Available |
| 21 | 21.3 | Reading for pleasure | Available |
| 21 | 21.4 | Playing a musical instrument | Available |
| 21 | 21.5 | Participation in trips | Available |
| 21 | 21.6 | Participation in organized or private social activity | Available |
| 21 | 21.7 | Watching TV | ABSENT |
| 21 | 21.8 | Playing with animals | ABSENT |
