# Supplementary material for: Greek Translation and Preliminary Test–Retest Reliability of the AQUA CHILD Questionnaire in Children With Developmental Disabilities
Source: Physiother Res Int. 2026 Oct 8;31(4):e70357. doi: 10.1002/pri.70357 (PMC13648008; doi:10.1002/pri.70357)
Supplement: Supplementary file 3 [file PRI-31-e70357-s002.docx]

# Supplementary Table S6A. Common-applicable-item sensitivity analysis

| **Domain** | **n** | **Common items per pair** | **ICC(A,1) [95% CI]** | **Mean difference [95% CI]** | **Raw p** | **Holm p** |
| --- | --- | --- | --- | --- | --- | --- |
| 2 | 47 | 2–3 | .910 [.843, .949] | −0.035 [−0.086, 0.015] | .168 | 1.000 |
| 3 | 47 | 1–2 | .968 [.944, .982] | 0.021 [−0.022, 0.064] | .323 | 1.000 |
| 4 | 47 | 3–4 | .959 [.926, .977] | −0.027 [−0.058, 0.005] | .096 | 1.000 |
| 5 | 47 | 2–3 | .898 [.824, .942] | −0.007 [−0.055, 0.041] | .767 | 1.000 |
| 6 | 47 | 2–3 | .934 [.851, .967] | 0.071 [0.030, 0.111] | < .001 | .019 |
| 7 | 47 | 5–5 | .943 [.900, .968] | 0.026 [−0.010, 0.061] | .160 | 1.000 |
| 8 | 47 | 3–4 | .960 [.930, .978] | −0.005 [−0.038, 0.027] | .743 | 1.000 |
| 9 | 47 | 8–9 | .940 [.892, .966] | 0.022 [−0.001, 0.044] | .058 | 1.000 |
| 10 | 47 | 4–6 | .916 [.855, .953] | 0.012 [−0.018, 0.043] | .418 | 1.000 |
| 11 | 47 | 3–4 | .932 [.882, .962] | 0.023 [−0.015, 0.061] | .229 | 1.000 |
| 12 | 47 | 7–8 | .885 [.804, .934] | −0.022 [−0.055, 0.011] | .185 | 1.000 |
| 13* | 47 | 5–5 | .963 [.935, .979] | −0.000 [−0.030, 0.030] | 1.000 | 1.000 |
| 14 | 47 | 3–4 | .902 [.831, .944] | −0.012 [−0.057, 0.032] | .574 | 1.000 |
| 15 | 47 | 4–5 | .931 [.879, .961] | 0.022 [−0.009, 0.054] | .156 | 1.000 |
| 16 | 47 | 7–8 | .937 [.890, .965] | −0.003 [−0.029, 0.023] | .837 | 1.000 |
| 17 | 47 | 2–4 | .921 [.863, .955] | 0.016 [−0.036, 0.068] | .537 | 1.000 |
| 18 | 47 | 3–3 | .906 [.836, .947] | 0.050 [−0.004, 0.104] | .070 | 1.000 |
| 19 | 47 | 3–4 | .945 [.904, .969] | 0.000 [−0.034, 0.034] | 1.000 | 1.000 |
| 20 | 47 | 4–5 | .949 [.911, .971] | −0.011 [−0.045, 0.024] | .541 | 1.000 |
| 21* | 47 | 5–6 | .942 [.898, .967] | −0.006 [−0.041, 0.028] | .708 | 1.000 |

This sensitivity analysis averages each participant’s items that are applicable at both visits; the same items are used at T1 and T2 within that pair. The range is 0–2, not 0–100. A single common applicable item is permitted here, so this is not equivalent to a validated domain scale or to the fixed-item primary sums. Missing CSV columns remain unavailable. Only Domain 6 remains significant after Holm adjustment. * Available-item subset.

# Supplementary Table S6B. Exact sign-flip sensitivity of the fixed-item paired differences

| **Domain** | **n** | **Non-zero differences** | **Exact sign-flip p** | **Holm p** |
| --- | --- | --- | --- | --- |
| 2 | 46 | 13 | .267 | 1.000 |
| 3 | 46 | 4 | .625 | 1.000 |
| 4 | 44 | 9 | .180 | 1.000 |
| 5 | 44 | 8 | 1.000 | 1.000 |
| 6 | 46 | 10 | .002 | .039 |
| 7 | 47 | 12 | .244 | 1.000 |
| 8 | 44 | 9 | 1.000 | 1.000 |
| 9 | 45 | 16 | .135 | 1.000 |
| 10 | 44 | 16 | .804 | 1.000 |
| 11 | 44 | 11 | .549 | 1.000 |
| 12 | 43 | 19 | .414 | 1.000 |
| 13* | 47 | 12 | 1.000 | 1.000 |
| 14 | 46 | 12 | 1.000 | 1.000 |
| 15 | 43 | 12 | .388 | 1.000 |
| 16 | 46 | 20 | 1.000 | 1.000 |
| 17 | 45 | 14 | .683 | 1.000 |
| 18 | 47 | 12 | .119 | 1.000 |
| 19 | 46 | 7 | 1.000 | 1.000 |
| 20 | 45 | 10 | 1.000 | 1.000 |
| 21* | 43 | 18 | .666 | 1.000 |

The two-sided test compares the absolute observed sum of differences with all equiprobable sign assignments to the observed non-zero magnitudes. Dynamic programming provides exact probabilities without Monte Carlo sampling. Its null requires symmetry/exchangeability of difference signs and is not assumption-free. Holm adjustment covers 20 tests. Domain 6: p = .001953125; Holm p = .0390625. * Available-item subset.
