# Supplementary material for: Greek Translation and Preliminary Test–Retest Reliability of the AQUA CHILD Questionnaire in Children With Developmental Disabilities
Source: Physiother Res Int. 2026 Oct 8;31(4):e70357. doi: 10.1002/pri.70357 (PMC13648008; doi:10.1002/pri.70357)
Supplement: Supplementary file 4 [file PRI-31-e70357-s003.docx]

# Table S5. Difference-distribution and magnitude diagnostics

| **Domain** | **Zero differences** | **Difference-on-mean slope** | **Raw p** | **Holm p** | **Magnitude–dispersion rho** | **Raw p** | **Holm p** |
| --- | --- | --- | --- | --- | --- | --- | --- |
| 2 | 33/46 | −0.086 | .186 | 1.000 | 0.005 | .972 | 1.000 |
| 3 | 42/46 | 0.014 | .725 | 1.000 | 0.198 | .186 | 1.000 |
| 4 | 35/44 | 0.058 | .189 | 1.000 | 0.036 | .816 | 1.000 |
| 5 | 36/44 | 0.007 | .926 | 1.000 | −0.114 | .460 | 1.000 |
| 6 | 36/46 | 0.080 | .106 | 1.000 | 0.283 | .057 | 1.000 |
| 7 | 35/47 | 0.039 | .442 | 1.000 | 0.116 | .439 | 1.000 |
| 8 | 35/44 | 0.042 | .361 | 1.000 | −0.079 | .609 | 1.000 |
| 9 | 29/45 | −0.021 | .682 | 1.000 | 0.088 | .563 | 1.000 |
| 10 | 28/44 | 0.079 | .203 | 1.000 | −0.158 | .306 | 1.000 |
| 11 | 33/44 | 0.091 | .095 | 1.000 | −0.043 | .782 | 1.000 |
| 12 | 24/43 | 0.178 | .022 | .443 | 0.039 | .802 | 1.000 |
| 13* | 35/47 | 0.038 | .352 | 1.000 | 0.054 | .717 | 1.000 |
| 14 | 34/46 | 0.100 | .127 | 1.000 | −0.094 | .534 | 1.000 |
| 15 | 31/43 | 0.093 | .103 | 1.000 | −0.138 | .379 | 1.000 |
| 16 | 26/46 | 0.032 | .562 | 1.000 | −0.069 | .650 | 1.000 |
| 17 | 31/45 | 0.020 | .773 | 1.000 | 0.087 | .572 | 1.000 |
| 18 | 35/47 | 0.012 | .857 | 1.000 | −0.079 | .596 | 1.000 |
| 19 | 39/46 | 0.023 | .653 | 1.000 | −0.019 | .899 | 1.000 |
| 20 | 35/45 | 0.067 | .085 | 1.000 | −0.129 | .400 | 1.000 |
| 21* | 25/43 | 0.017 | .758 | 1.000 | −0.240 | .122 | 1.000 |

Proportional-bias diagnostic: ordinary least-squares regression of T2−T1 on (T1+T2)/2. Dispersion diagnostic: Spearman correlation between the pair mean and |difference−mean difference|. Holm adjustment is separate within each 20-domain diagnostic family. Non-significant tests do not confirm homogeneous error, normality, or absence of proportional bias. * Available-item subset.
