# Supplementary material for: Greek Translation and Preliminary Test–Retest Reliability of the AQUA CHILD Questionnaire in Children With Developmental Disabilities
Source: Physiother Res Int. 2026 Oct 8;31(4):e70357. doi: 10.1002/pri.70357 (PMC13648008; doi:10.1002/pri.70357)
Supplement: Supplementary file 5 [file PRI-31-e70357-s004.docx]

# Table S1. Domain structure and available data

| **Domain** | **Published development categories** | **Numbered items on supplied forms** | **CSV variables** | **Analysed pairs** | **Absent variable** |
| --- | --- | --- | --- | --- | --- |
| 1. Swimming | 4 | 4 | 4 | 1.1–1.4: categorical | None |
| 2. Acquiring skills | 4 | 3 | 3 | 46 | None |
| 3. Focusing attention | 3 | 2 | 2 | 46 | None |
| 4. Solving problems | 4 | 4 | 4 | 44 | None |
| 5. Making decisions | 3 | 3 | 3 | 44 | None |
| 6. Undertaking a single task | 3 | 3 | 3 | 46 | None |
| 7. Managing one's own behaviour | 5 | 5 | 5 | 47 | None |
| 8. Conversation | 4 | 4 | 4 | 44 | None |
| 9. Changing basic body positions | 10 | 9 | 9 | 45 | None |
| 10. Maintaining a body position | 6 | 6 | 6 | 44 | None |
| 11. Hand and arm use | 5 | 4 | 4 | 44 | None |
| 12. Walking | 9 | 8 | 8 | 43 | None |
| 13. Moving around | 7 | 6 | 5 | 47 | 13.6 |
| 14. Moving around in different locations | 5 | 4 | 4 | 46 | None |
| 15. Caring for body parts | 6 | 5 | 5 | 43 | None |
| 16. Dressing | 9 | 8 | 8 | 46 | None |
| 17. Basic interpersonal interactions | 5 | 4 | 4 | 45 | None |
| 18. Informal relationships with peers | 4 | 3 | 3 | 47 | None |
| 19. Family relationships | 5 | 4 | 4 | 46 | None |
| 20. Engagement in play | 6 | 5 | 5 | 45 | None |
| 21. Recreation and leisure | 9 | 8 | 6 | 43 | 21.7, 21.8 |
| Total numbered/recorded items | 116 | 102 | 99 | Not additive | 13.6, 21.7, 21.8 |

Counts describe distinct source representations. They do not imply that every published category was a numbered item in the supplied form. No full-score result is inferred from the sum of the domain counts.
