# Supplementary material for: Greek Translation and Preliminary Test–Retest Reliability of the AQUA CHILD Questionnaire in Children With Developmental Disabilities
Source: Physiother Res Int. 2026 Oct 8;31(4):e70357. doi: 10.1002/pri.70357 (PMC13648008; doi:10.1002/pri.70357)
Supplement: Supplementary file 6 [file PRI-31-e70357-s005.docx]

# Table S4. Direct participant-level difference summaries and limits of agreement

| **Domain** | **n** | **Bias** | **SD diff.** | **Lower LoA [95% CI]** | **Upper LoA [95% CI]** | **Empirical 2.5%, 97.5%** | **Outside LoA** |
| --- | --- | --- | --- | --- | --- | --- | --- |
| 2 | 46 | −0.11 | 0.53 | −1.14 [−1.41, −0.87] | 0.92 [0.65, 1.19] | −1.00, 1.00 | 4/46 |
| 3 | 46 | 0.04 | 0.29 | −0.53 [−0.69, −0.38] | 0.62 [0.47, 0.77] | 0.00, 1.00 | 4/46 |
| 4 | 44 | −0.11 | 0.44 | −0.98 [−1.21, −0.75] | 0.75 [0.52, 0.99] | −1.00, 0.92 | 9/44 |
| 5 | 44 | −0.02 | 0.51 | −1.01 [−1.28, −0.75] | 0.97 [0.70, 1.23] | −1.00, 1.00 | 5/44 |
| 6 | 46 | 0.22 | 0.42 | −0.60 [−0.81, −0.39] | 1.03 [0.82, 1.25] | 0.00, 1.00 | 0/46 |
| 7 | 47 | 0.13 | 0.61 | −1.07 [−1.38, −0.76] | 1.33 [1.02, 1.64] | −1.00, 1.85 | 2/47 |
| 8 | 44 | −0.02 | 0.46 | −0.92 [−1.16, −0.68] | 0.87 [0.63, 1.11] | −1.00, 1.00 | 9/44 |
| 9 | 45 | 0.18 | 0.68 | −1.16 [−1.52, −0.81] | 1.52 [1.16, 1.87] | −1.00, 1.90 | 2/45 |
| 10 | 44 | 0.05 | 0.61 | −1.15 [−1.47, −0.83] | 1.24 [0.92, 1.56] | −1.00, 1.00 | 0/44 |
| 11 | 44 | 0.07 | 0.50 | −0.91 [−1.18, −0.65] | 1.05 [0.79, 1.31] | −1.00, 1.00 | 4/44 |
| 12 | 43 | −0.14 | 0.91 | −1.93 [−2.42, −1.45] | 1.65 [1.17, 2.14] | −2.00, 1.00 | 4/43 |
| 13* | 47 | 0.00 | 0.51 | −1.00 [−1.26, −0.74] | 1.00 [0.74, 1.26] | −1.00, 1.00 | 0/47 |
| 14 | 46 | −0.02 | 0.58 | −1.15 [−1.45, −0.86] | 1.11 [0.81, 1.40] | −1.00, 1.00 | 1/46 |
| 15 | 43 | 0.09 | 0.53 | −0.94 [−1.22, −0.66] | 1.12 [0.85, 1.40] | −1.00, 1.00 | 4/43 |
| 16 | 46 | −0.02 | 0.71 | −1.42 [−1.79, −1.06] | 1.38 [1.01, 1.74] | −1.00, 1.00 | 1/46 |
| 17 | 45 | 0.07 | 0.72 | −1.34 [−1.72, −0.97] | 1.48 [1.11, 1.85] | −1.00, 1.90 | 3/45 |
| 18 | 47 | 0.15 | 0.55 | −0.93 [−1.21, −0.65] | 1.23 [0.95, 1.51] | −1.00, 1.00 | 4/47 |
| 19 | 46 | 0.00 | 0.47 | −0.92 [−1.16, −0.68] | 0.92 [0.68, 1.16] | −1.00, 1.00 | 7/46 |
| 20 | 45 | 0.00 | 0.48 | −0.93 [−1.18, −0.69] | 0.93 [0.69, 1.18] | −1.00, 1.00 | 10/45 |
| 21* | 43 | −0.07 | 0.70 | −1.45 [−1.82, −1.08] | 1.31 [0.94, 1.68] | −1.00, 1.00 | 1/43 |

Bias = mean T2−T1. Nominal normal-theory LoA = bias ± 1.96 × SD of differences. Approximate model-based CI for a limit uses t(n−1, .975) × SD_diff × sqrt(1/n + 1.96²/[2(n−1)]). These CIs concern uncertainty in each limit, not an individual change or the mean. Empirical quantiles use linear interpolation and may lie between possible integer differences. * Available-item subset.

Discrete differences and many zeros make normal-theory 95% coverage unreliable. Observed coverage ranges from 77.8% to 100%. These are descriptive diagnostics, not validated individual clinical decision thresholds. Figures S1–S20 use the same paired observations and exclusions.
