# Supplementary material for: Greek Translation and Preliminary Test–Retest Reliability of the AQUA CHILD Questionnaire in Children With Developmental Disabilities
Source: Physiother Res Int. 2026 Oct 8;31(4):e70357. doi: 10.1002/pri.70357 (PMC13648008; doi:10.1002/pri.70357)
Supplement: Supplementary file 7 [file PRI-31-e70357-s007.docx]

# Supplementary Table S3. Item-level categorical agreement

All items use n = 47 pairs. Percentage CIs are exact binomial; kappa CIs are 10,000 paired-participant percentile bootstrap intervals (seed 8448979). Code 3 is a distinct nominal “not relevant” category in Domains 2–21. Applicable-only sensitivity excludes code 3 at either visit; in swimming, code 3 is a support category and is not excluded. No inference assumes 99 independent samples.

| **Item** | **Exact pairs** | **Agreement % [95% CI]** | **Kappa** | **Kappa 95% CI** | **T2 not relevant** | **Applicable pairs** | **Applicable kappa** |
| --- | --- | --- | --- | --- | --- | --- | --- |
| 1.1 | 45/47 | 95.7 [85.5, 99.5] | .949 | [.869, 1.000] | n/a | 47 | .949 |
| 1.2 | 43/47 | 91.5 [79.6, 97.6] | .899 | [.796, .975] | n/a | 47 | .899 |
| 1.3 | 45/47 | 95.7 [85.5, 99.5] | .950 | [.871, 1.000] | n/a | 47 | .950 |
| 1.4 | 46/47 | 97.9 [88.7, 99.9] | .974 | [.920, 1.000] | n/a | 47 | .974 |
| 2.1 | 40/47 | 85.1 [71.7, 93.8] | .759 | [.588, .901] | 1 | 46 | .784 |
| 2.2 | 44/47 | 93.6 [82.5, 98.7] | .900 | [.768, 1.000] | 0 | 47 | .900 |
| 2.3 | 43/47 | 91.5 [79.6, 97.6] | .863 | [.718, .967] | 0 | 47 | .863 |
| 3.1 | 43/47 | 91.5 [79.6, 97.6] | .865 | [.727, .967] | 1 | 46 | .895 |
| 3.2 | 46/47 | 97.9 [88.7, 99.9] | .967 | [.893, 1.000] | 0 | 47 | .967 |
| 4.1 | 44/47 | 93.6 [82.5, 98.7] | .899 | [.773, 1.000] | 1 | 46 | .930 |
| 4.2 | 44/47 | 93.6 [82.5, 98.7] | .896 | [.765, 1.000] | 0 | 47 | .896 |
| 4.3 | 43/47 | 91.5 [79.6, 97.6] | .864 | [.723, .967] | 1 | 46 | .893 |
| 4.4 | 45/47 | 95.7 [85.5, 99.5] | .929 | [.822, 1.000] | 1 | 46 | .963 |
| 5.1 | 41/47 | 87.2 [74.3, 95.2] | .801 | [.637, .933] | 2 | 45 | .856 |
| 5.2 | 44/47 | 93.6 [82.5, 98.7] | .891 | [.756, 1.000] | 1 | 46 | .924 |
| 5.3 | 44/47 | 93.6 [82.5, 98.7] | .891 | [.751, 1.000] | 0 | 47 | .891 |
| 6.1 | 42/47 | 89.4 [76.9, 96.5] | .832 | [.677, .964] | 1 | 46 | .860 |
| 6.2 | 42/47 | 89.4 [76.9, 96.5] | .817 | [.643, .960] | 0 | 47 | .817 |
| 6.3 | 44/47 | 93.6 [82.5, 98.7] | .885 | [.737, 1.000] | 0 | 47 | .885 |
| 7.1 | 45/47 | 95.7 [85.5, 99.5] | .928 | [.810, 1.000] | 0 | 47 | .928 |
| 7.2 | 44/47 | 93.6 [82.5, 98.7] | .887 | [.740, 1.000] | 0 | 47 | .887 |
| 7.3 | 42/47 | 89.4 [76.9, 96.5] | .835 | [.683, .966] | 0 | 47 | .835 |
| 7.4 | 42/47 | 89.4 [76.9, 96.5] | .833 | [.686, .964] | 0 | 47 | .833 |
| 7.5 | 44/47 | 93.6 [82.5, 98.7] | .897 | [.768, 1.000] | 0 | 47 | .897 |
| 8.1 | 46/47 | 97.9 [88.7, 99.9] | .966 | [.889, 1.000] | 0 | 47 | .966 |
| 8.2 | 39/47 | 83.0 [69.2, 92.4] | .748 | [.585, .900] | 2 | 45 | .797 |
| 8.3 | 44/47 | 93.6 [82.5, 98.7] | .898 | [.772, 1.000] | 1 | 46 | .929 |
| 8.4 | 45/47 | 95.7 [85.5, 99.5] | .931 | [.819, 1.000] | 0 | 47 | .931 |
| 9.1 | 41/47 | 87.2 [74.3, 95.2] | .805 | [.648, .935] | 0 | 47 | .805 |
| 9.2 | 42/47 | 89.4 [76.9, 96.5] | .829 | [.668, .963] | 0 | 47 | .829 |
| 9.3 | 45/47 | 95.7 [85.5, 99.5] | .932 | [.825, 1.000] | 0 | 47 | .932 |
| 9.4 | 43/47 | 91.5 [79.6, 97.6] | .867 | [.730, .968] | 1 | 46 | .896 |
| 9.5 | 44/47 | 93.6 [82.5, 98.7] | .902 | [.771, 1.000] | 0 | 47 | .902 |
| 9.6 | 42/47 | 89.4 [76.9, 96.5] | .830 | [.671, .963] | 0 | 47 | .830 |
| 9.7 | 44/47 | 93.6 [82.5, 98.7] | .901 | [.778, 1.000] | 1 | 46 | .931 |
| 9.8 | 44/47 | 93.6 [82.5, 98.7] | .902 | [.775, 1.000] | 0 | 47 | .902 |
| 9.9 | 45/47 | 95.7 [85.5, 99.5] | .933 | [.828, 1.000] | 0 | 47 | .933 |
| 10.1 | 46/47 | 97.9 [88.7, 99.9] | .968 | [.899, 1.000] | 0 | 47 | .968 |
| 10.2 | 41/47 | 87.2 [74.3, 95.2] | .791 | [.616, .931] | 0 | 47 | .791 |
| 10.3 | 42/47 | 89.4 [76.9, 96.5] | .826 | [.663, .962] | 1 | 46 | .855 |
| 10.4 | 43/47 | 91.5 [79.6, 97.6] | .865 | [.719, .967] | 0 | 47 | .865 |
| 10.5 | 44/47 | 93.6 [82.5, 98.7] | .892 | [.761, 1.000] | 1 | 46 | .925 |
| 10.6 | 41/47 | 87.2 [74.3, 95.2] | .799 | [.638, .933] | 2 | 45 | .855 |
| 11.1 | 43/47 | 91.5 [79.6, 97.6] | .858 | [.704, .966] | 0 | 47 | .858 |
| 11.2 | 41/47 | 87.2 [74.3, 95.2] | .807 | [.658, .934] | 2 | 45 | .861 |
| 11.3 | 46/47 | 97.9 [88.7, 99.9] | .965 | [.888, 1.000] | 0 | 47 | .965 |
| 11.4 | 43/47 | 91.5 [79.6, 97.6] | .862 | [.717, .966] | 1 | 46 | .892 |
| 12.1 | 44/47 | 93.6 [82.5, 98.7] | .899 | [.771, 1.000] | 1 | 46 | .930 |
| 12.2 | 44/47 | 93.6 [82.5, 98.7] | .896 | [.764, 1.000] | 1 | 46 | .928 |
| 12.3 | 44/47 | 93.6 [82.5, 98.7] | .893 | [.757, 1.000] | 0 | 47 | .893 |
| 12.4 | 43/47 | 91.5 [79.6, 97.6] | .863 | [.720, .967] | 1 | 46 | .893 |
| 12.5 | 41/47 | 87.2 [74.3, 95.2] | .780 | [.608, .926] | 0 | 47 | .780 |
| 12.6 | 42/47 | 89.4 [76.9, 96.5] | .836 | [.682, .966] | 0 | 47 | .836 |
| 12.7 | 39/47 | 83.0 [69.2, 92.4] | .722 | [.518, .886] | 0 | 47 | .722 |
| 12.8 | 45/47 | 95.7 [85.5, 99.5] | .932 | [.831, 1.000] | 1 | 46 | .965 |
| 13.1 | 44/47 | 93.6 [82.5, 98.7] | .895 | [.759, 1.000] | 0 | 47 | .895 |
| 13.2 | 45/47 | 95.7 [85.5, 99.5] | .935 | [.835, 1.000] | 0 | 47 | .935 |
| 13.3 | 42/47 | 89.4 [76.9, 96.5] | .827 | [.666, .964] | 0 | 47 | .827 |
| 13.4 | 45/47 | 95.7 [85.5, 99.5] | .927 | [.807, 1.000] | 0 | 47 | .927 |
| 13.5 | 45/47 | 95.7 [85.5, 99.5] | .933 | [.827, 1.000] | 0 | 47 | .933 |
| 14.1 | 42/47 | 89.4 [76.9, 96.5] | .802 | [.628, .955] | 1 | 46 | .832 |
| 14.2 | 42/47 | 89.4 [76.9, 96.5] | .821 | [.648, .962] | 0 | 47 | .821 |
| 14.3 | 46/47 | 97.9 [88.7, 99.9] | .966 | [.888, 1.000] | 0 | 47 | .966 |
| 14.4 | 43/47 | 91.5 [79.6, 97.6] | .859 | [.715, .966] | 0 | 47 | .859 |
| 15.1 | 41/47 | 87.2 [74.3, 95.2] | .770 | [.587, .923] | 2 | 45 | .829 |
| 15.2 | 41/47 | 87.2 [74.3, 95.2] | .800 | [.638, .934] | 0 | 47 | .800 |
| 15.3 | 45/47 | 95.7 [85.5, 99.5] | .926 | [.808, 1.000] | 0 | 47 | .926 |
| 15.4 | 42/47 | 89.4 [76.9, 96.5] | .836 | [.688, .965] | 1 | 46 | .864 |
| 15.5 | 45/47 | 95.7 [85.5, 99.5] | .931 | [.825, 1.000] | 1 | 46 | .964 |
| 16.1 | 41/47 | 87.2 [74.3, 95.2] | .803 | [.647, .933] | 1 | 46 | .830 |
| 16.2 | 43/47 | 91.5 [79.6, 97.6] | .863 | [.720, .967] | 0 | 47 | .863 |
| 16.3 | 46/47 | 97.9 [88.7, 99.9] | .959 | [.864, 1.000] | 0 | 47 | .959 |
| 16.4 | 47/47 | 100.0 [92.5, 100.0] | 1.000 | Not estimable* | 0 | 47 | 1.000 |
| 16.5 | 43/47 | 91.5 [79.6, 97.6] | .868 | [.730, .968] | 0 | 47 | .868 |
| 16.6 | 40/47 | 85.1 [71.7, 93.8] | .755 | [.574, .901] | 0 | 47 | .755 |
| 16.7 | 45/47 | 95.7 [85.5, 99.5] | .928 | [.815, 1.000] | 0 | 47 | .928 |
| 16.8 | 43/47 | 91.5 [79.6, 97.6] | .847 | [.681, .964] | 0 | 47 | .847 |
| 17.1 | 45/47 | 95.7 [85.5, 99.5] | .930 | [.820, 1.000] | 0 | 47 | .930 |
| 17.2 | 43/47 | 91.5 [79.6, 97.6] | .873 | [.743, .968] | 1 | 46 | .901 |
| 17.3 | 40/47 | 85.1 [71.7, 93.8] | .766 | [.582, .923] | 0 | 47 | .766 |
| 17.4 | 38/47 | 80.9 [66.7, 90.9] | .715 | [.540, .871] | 2 | 45 | .763 |
| 18.1 | 42/47 | 89.4 [76.9, 96.5] | .840 | [.691, .967] | 0 | 47 | .840 |
| 18.2 | 42/47 | 89.4 [76.9, 96.5] | .830 | [.672, .965] | 0 | 47 | .830 |
| 18.3 | 44/47 | 93.6 [82.5, 98.7] | .894 | [.758, 1.000] | 0 | 47 | .894 |
| 19.1 | 45/47 | 95.7 [85.5, 99.5] | .932 | [.826, 1.000] | 0 | 47 | .932 |
| 19.2 | 44/47 | 93.6 [82.5, 98.7] | .880 | [.725, 1.000] | 0 | 47 | .880 |
| 19.3 | 42/47 | 89.4 [76.9, 96.5] | .830 | [.675, .964] | 1 | 46 | .858 |
| 19.4 | 46/47 | 97.9 [88.7, 99.9] | .959 | [.859, 1.000] | 0 | 47 | .959 |
| 20.1 | 44/47 | 93.6 [82.5, 98.7] | .896 | [.761, 1.000] | 0 | 47 | .896 |
| 20.2 | 40/47 | 85.1 [71.7, 93.8] | .763 | [.593, .901] | 1 | 46 | .788 |
| 20.3 | 46/47 | 97.9 [88.7, 99.9] | .968 | [.904, 1.000] | 1 | 46 | 1.000 |
| 20.4 | 44/47 | 93.6 [82.5, 98.7] | .903 | [.777, 1.000] | 0 | 47 | .903 |
| 20.5 | 45/47 | 95.7 [85.5, 99.5] | .934 | [.831, 1.000] | 0 | 47 | .934 |
| 21.1 | 44/47 | 93.6 [82.5, 98.7] | .900 | [.771, 1.000] | 0 | 47 | .900 |
| 21.2 | 45/47 | 95.7 [85.5, 99.5] | .936 | [.834, 1.000] | 0 | 47 | .936 |
| 21.3 | 40/47 | 85.1 [71.7, 93.8] | .752 | [.562, .900] | 0 | 47 | .752 |
| 21.4 | 41/47 | 87.2 [74.3, 95.2] | .804 | [.638, .934] | 2 | 45 | .859 |
| 21.5 | 42/47 | 89.4 [76.9, 96.5] | .829 | [.677, .962] | 2 | 45 | .888 |
| 21.6 | 40/47 | 85.1 [71.7, 93.8] | .762 | [.580, .902] | 0 | 47 | .762 |

* With perfect observed agreement, the empirical paired bootstrap is degenerate at kappa = 1 and does not provide informative uncertainty. The exact binomial agreement interval is reported instead; perfect sample agreement does not establish perfect population agreement. Blank response cells: zero in the 99 available variables. Code-3 responses are not blank cells.
